# Imidazolium Chloride Ionic Liquid Mixtures as Separating Agents: Fuel Processing and Azeotrope Breaking

**DOI:** 10.1021/acs.energyfuels.2c01724

**Published:** 2022-07-26

**Authors:** Sérgio M. Vilas-Boas, Mónia A. R. Martins, Fábio R. Tentor, Gabriel Teixeira, Juliana G. Sgorlon, João A. P. Coutinho, Olga Ferreira, Simão P. Pinho

**Affiliations:** † Centro de Investigação de Montanha (CIMO), Instituto Politécnico de Bragança, Campus de Santa Apolónia, 5300-253 Bragança, Portugal; ‡ CICECO-Aveiro Institute of Materials, Department of Chemistry, University of Aveiro, 3810-193 Aveiro, Portugal; § Federal University of Technology of Paraná-UTFPR, Rua Marcílio Dias, 635, Apucarana, 86812-460 Parana, Brazil

## Abstract

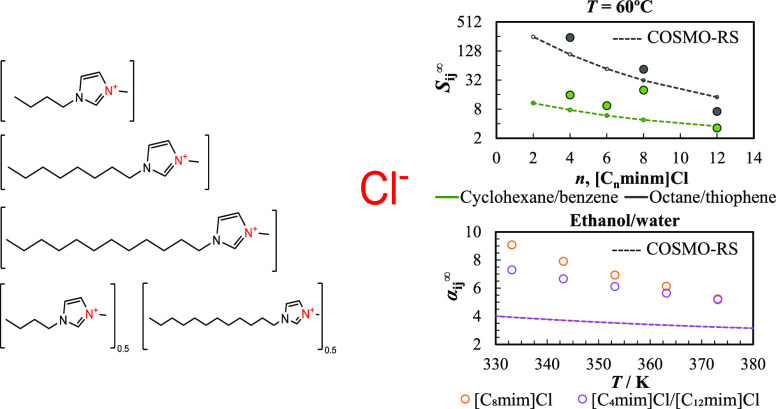

Relevant chemical separations for the petrochemical and chemical industries include the removal of aromatic hydrocarbons from aliphatics, the desulfurization and denitrification of fuels, and the separation of azeotropic mixtures containing alkanols. In an attempt to contribute to the development of novel technologies, the potentialities of imidazolium chloride ionic liquid (IL) mixtures as separation agents were investigated. Selectivities, capacities, and solvent performance indices were calculated through the activity coefficients at infinite dilution of organic solutes and water in the imidazolium chloride IL: [C_8_mim]Cl, [C_12_mim]Cl, and the equimolar mixture of [C_4_mim]Cl and [C_12_mim]Cl. Results show that the imidazolium chloride IL might be appropriately tailored for specific purposes, in which an increase in the proportion of cations containing larger alkyl chains tends to increase the overall affinity with organic solutes. The IL designer solvent concept was explored by comparing the IL equimolar mixture results with the intermediary [C_8_mim]Cl. The COSMO-RS thermodynamic model was also applied, showing it to be a promising tool for a fast qualitative screening of potential separation agents for specific separation processes.

## Introduction

The separation of hydrocarbons from crude oil, alkenes from alkanes, and benzene derivatives from analogues are of utmost importance and, as stated by Sholl and Lively,^1^ mandatory “to change the world”. Alternative processes without the use of high temperatures, as in distillation, would reduce costs and energy demands and consequently emissions to the environment, lowering pollution.^1^ Technologies based on the molecules’ chemical properties or size, such as membranes, have been attempted,^2,3^ however, are still underdeveloped and difficult to scale up, inhibiting industrial applications.

One of the most promising approaches currently explored by academic researchers is solvent extraction, with the traditional sulfolanes, esters, or alcohols; or with neoteric solvents as the well-known ionic liquids (IL).

ILs are a diverse class of complex organic salts exhibiting desirable properties, such as negligible volatility at the operating conditions, excellent thermal and chemical stability, high selectivity, and low flammability.^4,5^ Their unique nature and appealing properties have allowed exceptional achievements in many different fields in the last few decades, including electrochemistry, separation processes, biotechnology, metal processing, and as alternative solvents to the traditional volatile organic compounds (VOCs) by reducing the emission of hazardous pollutants.^6−8^

The potential of ILs as separation agents in chemical processes is enormous and, despite the many works performed, is still in its infancy. IL potential in chemical separation processes can be exploited using screening methods, thus avoiding extensive experimental liquid–liquid extractions. Solute–solvent interactions and useful separation parameters, such as selectivity, capacity, and partition coefficients,^9,10^ can be derived from the activity coefficients at infinite dilution.

Domańska’s and Mutelet’s research groups have extensively investigated the role of IL as “green” solvents in industrial separation processes for the extraction of sulfur- and nitrogen-containing compounds from fuels and as entrainers in the azeotrope breaking, which can replace commonly used VOCs and reduce the cost of industrial processes. More recently, Klimenko et al.^11^ evaluated a large number of available selectivities calculated from γ_13_^∞^ data, concluding that around 80% of IL identified as “selective” solvents by *S*_*ij*_^∞^ data are actually selective for liquid–liquid extraction processes.

Although the interest in ionic liquids has exploded throughout the scientific community in the last two decades,^12^ most of the reported research has focused on the applications of pure ionic liquids,^13,14^ and the investigation of mixtures of ionic liquids is still emerging.^15,16^ The use of those mixtures as entrainers in distillation or liquid–liquid extraction processes remains unexplored, probably due to the lack of available equilibrium properties between these mixtures and the target compounds, crucial for the designing and optimization of separation processes.

In this work, the potential of imidazolium chloride-based ionic liquid mixtures as separation agents for specific separation processes, namely, the desulfurization and denitrification fuels, the removal of aliphatic hydrocarbons from aromatics, and the breaking of azeotropic behavior, were evaluated. The activity coefficients at infinite dilution (γ_13_^∞^) of 28 organic solutes and water were measured in two pure chloride ILs, [C_8_mim]Cl and [C_12_mim]Cl, and in the equimolar mixture of [C_4_mim]Cl and [C_12_mim]Cl, by inverse gas chromatography in the temperature range of (333.15–423.15) K. From the experimental γ_13_^∞^ data, the most relevant parameters for the separation problems mentioned above were calculated. Lastly, the ability to customize the separation parameters was assessed using the predictive Conductor-like Screening Model for Real Solvents (COSMO-RS) model^17−19^ due to its well-recognized capabilities in predicting thermodynamic data, including γ_13_^∞^.^20−23^

## Experimental Section

Ionic liquids were dried under vacuum (at 1 Pa and 298.15 K), under continuous stirring, for at least 48 h before being used. Organic solutes were used as received from the supplier. IL and organic solutes’ chemical structures and properties are displayed in Tables S1 and S2, respectively, of Section S1 of the Supporting Information. The experimental procedure for the column packing and for the chromatographic experiments is described in detail in our previous works^24−27^ and summarized in Section S1 of the Supporting Information. The activity coefficients at infinite dilution, gas–liquid partition coefficients, and separation factor rational and equations are presented in Section S2 of the Supporting Information.

## COSMO-RS

The Conductor-like Screening Model for Real Solvents (COSMO-RS) combines quantum chemical treatment with statistical thermodynamics to predict thermodynamic properties.^21,22^ Briefly, the model can predict the thermodynamic data of a pure compound or multicomponent system, requiring only optimized molecular geometry, energy, and polarization charge density, σ, of each involved molecule.^21,28^ COSMO-RS has been extensively applied to estimate γ_13_^∞^ data of organic solutes in liquid solvents,^29,30^ particularly in IL.^20,26,31−33^

In this work, COSMO-RS predictions were performed through the COSMOtherm 2021 software^34^ in two quantum chemical levels, namely, the BP_TZVP_21 and BP_TZVPD_FINE_21 parametrizations. The input COSMO files used in the calculations were already available in the COSMOtherm database, except for [C_12_mim]Cl. For this IL, the calculations to obtain the optical energetic state were performed in the software TmoleX 4.5.3N using both GAS-COSMO-BP-TZVP and GAS-COSMO-BP-TZVPD-FINE templates. To perform the COSMO-RS calculations, the ILs were described as equimolar and electroneutral mixtures of the cations and anions.

## Results

### Activity Coefficients at Infinite Dilution

The activity coefficients at infinite dilution, γ_13_^∞^, measured in this work are listed in Table S3 of the Supporting Information. For the aliphatic and aromatic hydrocarbons, esters, ethers, and ketones, γ_13_^∞^ rank in the following order: [C_8_mim]Cl > [C_4_mim]Cl/[C_12_mim]Cl mixture > [C_12_mim]Cl. For thiophene, water, and most of the alcohols, there is an inversion in the ranking between the equimolar mixture and [C_8_mim]Cl. In organic solutes containing nitrogen—acetonitrile and pyridine—the highest γ_13_^∞^ occurs in [C_12_mim]Cl and in the equimolar mixture, respectively.

Regarding the [C_4_mim]Cl/[C_12_mim]Cl mixture, the activity coefficients are generally slightly lower than the values registered in [C_8_mim]Cl, though the difference is more substantial for low polar solutes, such as alkanes and cycloalkanes. The numerical difference is typically lower than 10% for most of the solutes, except for aliphatic hydrocarbons, ethylbenzene, and diethyl ether—solutes with γ_13_^∞^ > 10. To the best of our knowledge, to date there is only one work reporting experimental γ_13_^∞^ in mixtures of ionic liquids,^15^ where the authors investigated 11 organic solvents in the equimolar mixture of [C_4_mim]Cl and [C_4_mim][Tf_2_N]. Thus, the effect of combining two different cations in a binary mixture of IL with a common anion is first evaluated in this work.

In general, activity coefficients of alcohols and water in the three ILs studied are significantly lower than 1, indicating favorable solute–solvent interactions, which can be attributed to the hydrogen bond formation. For thiophene, pyridine, and acetonitrile, the γ_13_^∞^ values are close to unity, whereas positive deviations from ideality are observed for the other organic solutes, being more pronounced for aliphatic hydrocarbons and diethyl ether. In the three solvents under study, the highest γ_13_^∞^ were obtained for alkanes, though the γ_13_^∞^ values significantly decrease as the cation alkyl chain length of the IL increases, from [C_8_mim] to [C_12_mim]. Larger alkyl chains attached to the IL cation are expected to strengthen dispersive solute–solvent interactions, which play a key role in the dissolution behavior of apolar solutes, such as alkanes.^35−38^

To further explore the cation effects in the solvation behavior of the different chemical families in the chloride-based IL, and to compare more effectively the results for [C_4_mim]Cl/[C_12_mim]Cl equimolar mixture and [C_8_mim]Cl, the ln(γ_13_^∞^) versus 1000/*T* were plotted for all the studied solutes—Figure S1 of the Supporting Information. For comparison purposes, the γ_13_^∞^ data measured in [C_4_mim]Cl in a previous work from our group^24^ are also included. In general, good correlations between the ln(γ_13_^∞^) and temperature are observed for the different chemical families in the imidazolium chloride IL. Besides, the overall tendency of increasing the γ_13_^∞^ values by decreasing the cation alkyl chain length is also observed for [C_4_mim]Cl.^24^ Nevertheless, the effects of the cation alkyl chain length are less evident in strong polar solutes, such as water, methanol, and ethanol, in which the hydrogen bond formation dominates over the dispersive forces.

### Gas–Liquid Partition Coefficients

The gas–liquid partition coefficient, *K*_L_, compares the distribution of the solute partitioning between the ionic liquid and the gas phases, providing insights into the suitability of ionic liquid separation processes. To better understand the IL cation effect on the *K*_L_ values as well as to evaluate the relationship of this property with the structure and functionality of the solutes, the *K*_L_ values were calculated and are presented in Table S4 and Figure S2 of the Supporting Information along with the data retrieved from the literature for [C_4_mim]Cl,^24^ at 373.15 K. The *K*_L_ calculation procedure is also described in the Supporting Information, and the necessary density values are available in Tables S5 and S6.

As shown in Figure S2, at a fixed temperature, the partition coefficients increase with the solute’s number of carbons for alkanes, cycloalkanes, aromatic hydrocarbons, ketones, and some esters. The highest values are observed for strongly polar alcohols and water indicating larger affinities of these solutes to the liquid phases. On the other hand, apolar aliphatic hydrocarbons and diethyl ether present the lowest values of *K*_L_, especially with the IL [C_4_mim]Cl as observed before.^24,25^ This indicates the potential of imidazolium chloride IL to separate aliphatic hydrocarbons from polar alcohols and water.

### Limiting Partial Molar Excess Properties

To further investigate the solute-IL affinity, the limiting partial molar excess properties Gibbs energy (*G̅*_*m*_^*E*^), enthalpy (*H̅*_*m*_^*E*^), and entropy (*S̅*_*m*_^*E*^) were calculated for the studied solutes in the imidazolium chloride ionic liquids, at 373.15 K, and the results are presented in Table S7 and Figure S3 of the Supporting Information (the calculation procedure is presented in the Supporting Information).

A detailed discussion about the distribution of the partial molar thermodynamic functions of the studied solutes between the four regions presented in Figure S3 is provided in Section S3 of the Supporting Information, as well as insights on the solute-IL interactions.

Overall results demonstrate that the cation nature and the alkyl chain length play a relevant role in the solutes’ solvation process for ILs with a common anion, as shown before.^27,39^ Alcohols and water solvation in the imidazolium chloride IL is thermodynamically favorable. Besides, there is a dominant enthalpic effect on the solvation of these polar protic solutes. For alkanes and diethyl ether, the solvation in the studied IL is highly unfavorable. Regarding the aromatic hydrocarbons, these are distributed within region III, where the entropic effect is dominant. Thiophene and the nitrogen compounds have similar enthalpic and entropic contributions.

Additionally, the overall patterns of the partial molar excess properties in [C_8_mim]Cl and in the equimolar [C_4_mim]Cl/[C_12_mim]Cl mixture are similar, which is in accordance to the evidences found for γ_13_^∞^ and *K*_L_ in these IL.

### Separation Factors

Selectivities, *S*_*ij*_^∞^, and capacities, *k*_*j*_^∞^, are valuable parameters to evaluate the performance of a solvent as a separation agent to a target homogeneous binary mixture. Low *S*_*ij*_^∞^ values lead to poor separation efficiencies, while a low *k*_*j*_^∞^ is a consequence of poor solute–solvent affinity, suggesting that large amounts of solvents might be required to carry out the separation.^40,41^ Therefore, the search for an appropriate solvent is often challenging, and a reasonable balance between selectivities and capacities needs to be found, which might be fairly described by the solvent performance index (*Q*_*ij*_^∞^). In this work, the potential of imidazolium chloride IL, in terms of *S*_*ij*_^∞^ and *Q*_*ij*_^∞^, as separation agents for some significant separation problems, namely, the removal of aromatics from aliphatic hydrocarbons, the removal of contaminants from fuels, and the separation of azeotropic mixtures, is evaluated.

### Removal of Aromatics from Aliphatic Hydrocarbons

The separation of aromatic hydrocarbons from aliphatic hydrocarbons is usually challenging due to their close boiling points and the possible formation of azeotropic mixtures.^42^ Traditionally, these separations are carried out by liquid–liquid extraction, extractive or azeotropic distillation, using polar solvents, such as sulfolane, *n*-formyl morpholine (NFM), and *n*-methyl pyrrolidone (NMP). In this work, two of the most relevant aromatic/aliphatic separation problems in the petrochemical industry are addressed: the octane/benzene and the cyclohexane/benzene mixtures.

**Figure 1:**
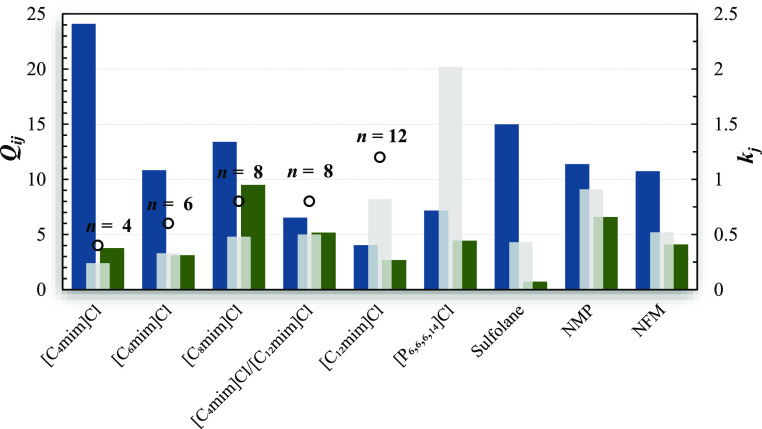
Comparison between the solvent performance index (dark colored bars) and capacities (light gray colored bars) at infinite dilution for the separation of octane/benzene (blue) and cyclohexane/benzene (green) at 333.15 K in [C_4_mim]Cl,^24^ [C_6_mim]Cl,^43^ [C_8_mim]Cl (this work), equimolar [C_4_mim]Cl/[C_12_mim]Cl mixture (this work), [C_12_mim]Cl (this work), [P_6,6,6,14_]Cl,^26^ sulfolane,^45^ NFM,^46,47^ and NMP.^46,47^ The open circles represent the number of carbons *n* in [C_*n*_mim]^+^.

The separation parameters for those mixtures with the investigated chloride-based IL are presented in Figure 1 and listed in Table S8, along with data available for other chloride-based ILs^24,26,43^ and organic solvents commonly used in industry.^44−47^ The *k*_*j*_^∞^ and *Q*_*ij*_^∞^ values for the cyclohexane/benzene with NMP and NFM included in Figure 1 are the average of the literature data.^46,47^

For the imidazolium chloride-based ILs and the octane/benzene mixture, *k*_*j*_^∞^ slightly increases as *n* increases, while the opposite trend is observed for *Q*_*ij*_^∞^ (except for [C_6_mim]Cl^43^). Therefore, the use of imidazolium chloride ionic liquids with shorter alkyl chain lengths as separation agents is recommended, with [C_4_mim]Cl delivering a solvent performance index of at least 1.6 higher than the traditional organic solvents sulfolane (*Q*_*ij*_^∞^ = 14.98),^45^ NMP (*Q*_*ij*_^∞^ = 11.38),^47^ and NMF (*Q*_*ij*_^∞^ = 10.74).^47^ Alternatively, [C_8_mim]Cl and the equimolar mixture could also be explored since they offer solvent performance indices similar to the abovementioned organic solvent values.

Regarding the cyclohexane/benzene mixture, although the same pattern described above is observed for the capacities, *Q*_*ij*_^∞^ do not follow any straightforward trend, being the highest value registered for [C_8_mim]Cl (9.50), that provides a solvent performance index of at least 20% higher than NMP (*Q*_*ij*_^∞^ = 7.94;^46^*Q*_*ij*_^∞^ = 5.23^47^), the best option among the three traditional organic solvents. The equimolar mixture of [C_4_mim]Cl and [C_12_mim]Cl (*Q*_*ij*_^∞^ = 5.17) also delivers a reasonable performance for the separation of cyclohexane from benzene, being superior to values found for NFM (*Q*_*ij*_^∞^ = 4.45;^46^*Q*_*ij*_^∞^ = 3.75^47^) and sulfolane (*Q*_*ij*_^∞^ = 0.72).

In both cases, [C_12_mim]Cl is the least promising option (*Q*_*ij*,octane/benzene_^∞^ = 4.03, *Q*_*ij*,cyclohexane/benzene_^∞^ = 2.68) among the studied ILs. The phosphonium-based [P_6,6,6,14_]Cl presents the highest capacity for benzene (*k*_*j*_^∞^ = 2.02),^26^ as a consequence of its stronger affinity with this aromatic organic solute. However, the low observed selectivities lead to modest *Q*_*ij*_^∞^ for both separations, often lower than those registered for the traditional organic solvents or imidazolium ILs with shorter cation carbon chains (C_4_–C_8_).

### Desulfurization and Denitrification of Fuels

**Figure 2:**
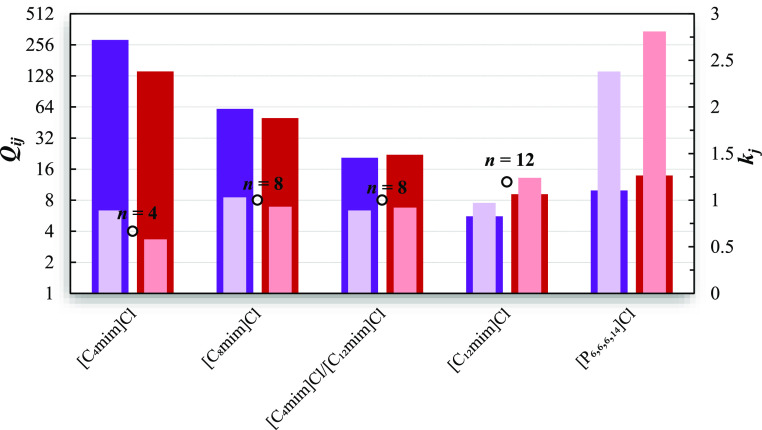
Comparison between the solvent performance index (dark colored bars) and capacities (light colored bars) at infinite dilution for the separation of octane/pyridine (purple) and octane/thiophene (red) at 333.15 K in [C_4_mim]Cl,^24^ [C_8_mim]Cl (this work), equimolar [C_4_mim]Cl/[C_12_mim]Cl mixture (this work), [C_12_mim]Cl (this work), and [P_6,6,6,14_]Cl.^26^ The open circles represent the number of carbons *n* in [C_*n*_mim]^+^.

The presence of sulfur- and nitrogen-containing compounds in petroleum refining processes is undesirable due to the deactivation of some catalysts and possible equipment corrosion.^48,49^ As the environmental regulations aiming at limiting the number of heterocyclic compounds in fuels increase,^49,50^ refineries are constantly searching for feasible and efficient methods to decrease the amount of these contaminants in fuels. Here, the potential of chloride-based ionic liquids for the separation of octane/pyridine and octane/thiophene mixtures through the solvent performance indices and capacities at 333.15 K is evaluated. The results are presented in Figure 2 and Table S9 of the Supporting Information.

[C_4_mim]Cl presents the highest solvent performance indices and the lowest capacities for both separations; and while larger IL cation alkyl chains improve the capacity, they induce considerably lower selectivities. As can be seen in Table S9, an increment from C4 to C12 in the IL cation alkyl chain leads to *k*_*j*_^∞^ and *Q*_*ij*_^∞^ values 1.09 times higher and 0.02 lower for octane/pyridine and 2.14 times higher and 0.06 lower for octane/thiophene, respectively. Moreover, similar to the benzene/octane case, [C_8_mim]Cl and [C_4_mim]Cl/[C_12_mim]Cl deliver intermediate *Q*_*ij*_^∞^ values for the removal of pyridine and thiophene from fuels in comparison with pure [C_4_mim]Cl and [C_12_mim]Cl.

Once more, interesting capacities are found when using the phosphonium IL; however, the much lower selectivities result in moderate *Q*_*ij*_^∞^ values. In the literature, the better performance of imidazolium-based IL over phosphonium IL for thiophene/alkane and pyridine/alkane mixtures have already been reported.^26,51^

Although all the ILs evaluated present reasonable *Q*_*ij*_^∞^ and *k*_*j*_^∞^ values, the very high solvent performance indices found in [C_4_mim]Cl suggest that this IL is the most promising option to be exploited in the denitrification and desulfurization of fuels.

### Separation of Azeotropic Mixtures of Alcohols

**Figure 3:**
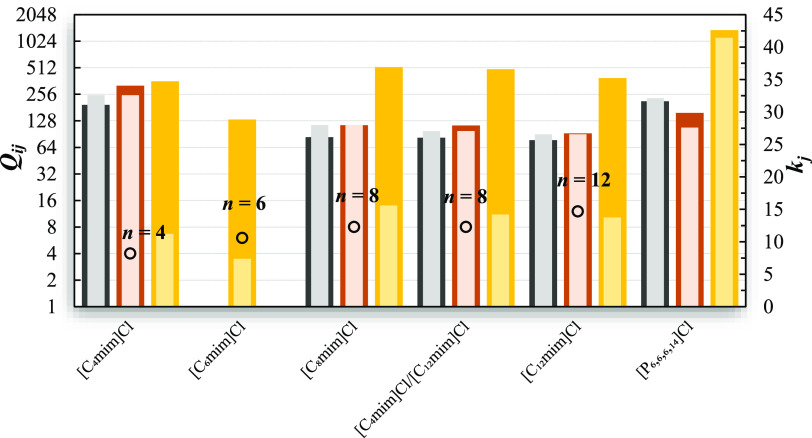
Comparison between the solvent performance index (dark colored bars) and capacities (light colored bars) at infinite dilution for the separation of ethanol/water (gray), 2-propanol/water (orange), and acetone/methanol (yellow) mixtures at 333.15 K in [C_4_mim]Cl,^24^ [C_6_mim]Cl,^43^ [C_8_mim]Cl (this work), equimolar [C_4_mim]Cl/[C_12_mim]Cl mixture (this work), [C_12_mim]Cl (this work), and [P_6,6,6,14_]Cl.^26^ The open circles represent the number of carbons *n* in [C_*n*_mim]^+^.

Several liquid mixtures used in industry present low relative volatility, preventing their efficient separation by simple distillation. Consequently, extractive or azeotropic distillation is usually implemented by adding a separation agent to the mixture, increasing considerably the relative volatility.^11,52^ Ionic liquids appear as attractive options due to their highly selective and nonvolatile nature,^53,54^ showing promising results for breaking water/ethanol and water/THF azeotropes.^55^ Therefore, the potentialities of the studied IL were assessed for the separation of three industrial relevant azeotropic mixtures containing alkanols: ethanol/water, 2-propanol/water, and acetone/methanol. The *S*_*ij*_^∞^ and *k*_*j*_^∞^ values obtained in this work are presented in Figure 3 and listed in Table S10 of the Supporting Information, along with the data found in the literature for other chloride-based ILs.^24,26,43^

As shown in Figure 3, excellent solvent performance indices (*Q*_*ij*_^∞^ ≥ 77.35) and capacities (*k*_*j*_^∞^ ≥ 7.37) are registered for the addressed azeotropic mixtures using the chloride-based ILs. These notable values might be attributed to the strong hydrogen bonding acceptor character of the chloride anion,^56,57^ enabling the formation of strong hydrogen bonds with alcohols and water.

Regarding the acetone/methanol mixture, very good solvent performance indices (*Q*_*ij*_^∞^ > 132.51) were obtained in the addressed ILs, being the highest value registered for [P_6,6,6,14_]Cl (*Q*_*ij*_^∞^ = 1363.96).^26^ The appealing *Q*_*ij*_^∞^ values combined with the good capacities (*k*_*ij*_^∞^ ≥ 7.37) suggest that the chloride ILs are great options for breaking this important azeotrope. For alcohol/water mixtures, attractive solvent performance indices (77 < *Q*_*ij*_^∞^ < 320) were also observed, being the highest *Q*_*ij*_^∞^ value registered for 2-propanol/water with [C_4_mim]Cl. [P_6,6,6,14_]Cl and [C_4_mim]Cl are the best options when treating the ethanol/water mixture. Moreover, [C_8_mim]Cl, [C_12_mim]Cl, and [C_4_mim]Cl/[C_12_mim]Cl equimolar mixture perform similarly in the separation of the studied azeotropes.

### COSMO-RS Predictions

#### Selectivities and Capacities

**Figure 4:**
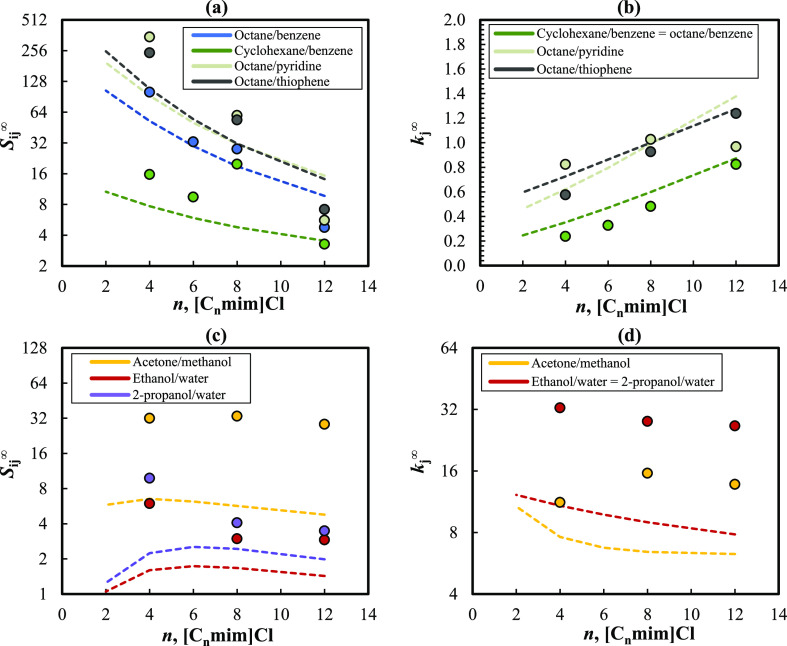
Comparison between the experimental and estimated *S*_*ij*_^∞^ and *k*_*j*_^∞^ values for the addressed separation problems in imidazolium chloride IL with different cation alkyl chain lengths. The filled symbols represent the experimental data obtained in this work and available in the literature,^24,43^ and the dotted lines depict the COSMO-RS results. Predictions from (a,b) were carried out at the TZVP-FINE level, while those from (c,d) were performed with the TZVP parametrization set.

In Figure 4, the selectivities and capacities (at 333.15 K) obtained with COSMO-RS for the separation problems discussed above in imidazolium chloride ionic liquids are compared with the available experimental data. For azeotropic mixtures, the values obtained with the TZVP parameterization are displayed, whereas the TZVPD-FINE quantum chemical level is used for the remaining separations according with the data quality, as discussed below. The predicted values are listed in Table S11 of the Supporting Information for both TZVP and TZVPD-FINE parametrization sets.

The results illustrated in Figure 4 show that COSMO-RS can efficiently capture the selectivity and capacity trends for the aliphatic/benzene, octane/pyridine, and octane/thiophene separation problems in the different imidazolium chloride-based ionic liquids using the TZVPD-FINE parametrization set. For these mixtures, the model demonstrates that an increase in the cation alkyl chain length leads to, respectively, a decrease and an increase in *S*_*ij*_^∞^ and *k*_*j*_^∞^ values, which is confirmed by the available experimental data. Although the predicted selectivities are often lower than the experimental values (except for [C_12_mim]Cl), the differences follow a systematic pattern for most cases. Besides, COSMO-RS provides excellent *k*_*j*_^∞^ estimations for the octane/contaminant mixtures, achieving a global average relative deviation (ARD) between the experimental and calculated *k*_*j*_^∞^ of 21%. For these separations, the predictions obtained with the TZVP basis set were considerably less accurate, particularly for selectivities where the trend with the IL cation size evidenced by the experimental data was not captured. It is noteworthy that the differences in the predicted *S*_*ij*_^∞^ and *k*_*j*_^∞^ values obtained for the mixtures containing alkanes with TZVP and TZVPD-FINE levels appear to be more pronounced for the IL with shorter cation alkyl chains, particularly for [C_2_mim]Cl and [C_4_mim]Cl.

Regarding azeotropic mixtures, the differences between the predicted selectivities using TZVP and TZVPD-FINE levels are lower than in the previous cases. Nevertheless, significantly better capacities were obtained using the TZVP basis set because of the lower γ_13_^∞^ estimated for water and alcohols, which are much closer to the experimental values. This is in line with the work of Paduszyński,^20^ whose conclusion was that a better description of the γ_13_^∞^ data of polar and associating solutes is obtained with the TZVP level, whereas the TZVPD-FINE performs better for mixtures containing aliphatic hydrocarbons.

**Figure 5:**
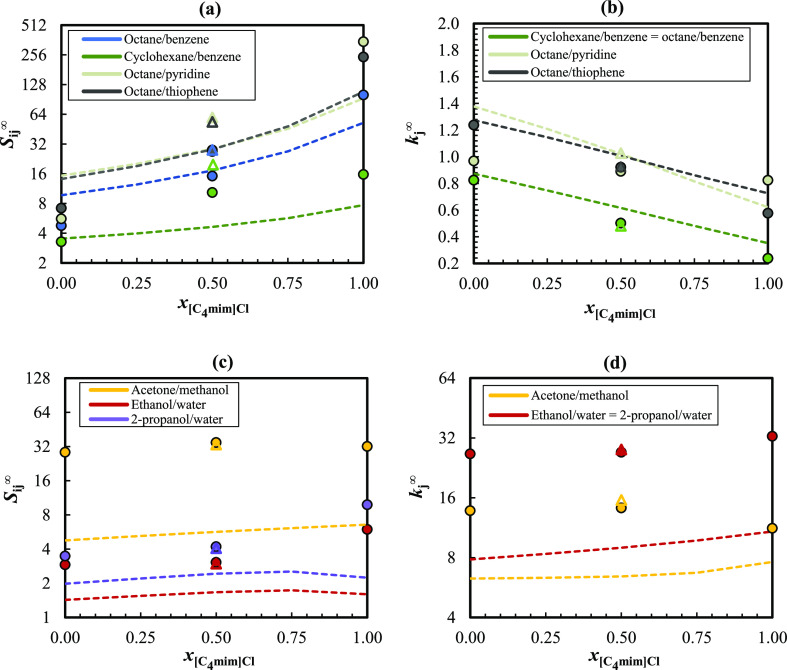
Comparison between the experimental and estimated selectivities and capacities of the studied separation problems in IL mixtures with different molar proportions of [C_4_mim]Cl and [C_12_mim]Cl. The filled circles correspond to the experimental data, and the dotted lines depict the COSMO-RS results. Predictions from (a,b) were carried out at the TZVP-FINE level, while those from (c,d) were performed with the TZVP parametrization set. The open triangles correspond to experimental separation factors obtained using pure [C_8_mim]Cl.

Aiming at evaluating the capabilities of COSMO-RS to describe the selectivities and capacities using ionic liquid mixtures, predictions were carried for the separation problems under study, in mixtures of [C_4_mim]Cl and [C_12_mim]Cl with different molar proportions, using both TZVP and TZVPD-FINE parametrization levels. Again, the best predictions for the azeotropic mixtures were obtained at the TZVP level, whereas all the other mixtures are more accurately described with TZVPD-FINE. The results are displayed in Figure 5 and Table S12 of the Supporting Information. For comparison purposes, the experimental separation factors obtained in pure [C_8_mim]Cl were also included in Figure 5.

Like observed before for pure ILs, COSMO-RS with the TZVPD-FINE level adequately represents the *S*_*ij*_^∞^ and *k*_*j*_^∞^ trends of binary mixtures containing alkanes and its variation with the IL mixture composition. Regarding selectivities, the model generally underestimates the values in pure [C_12_mim]Cl and overestimates them in pure [C_4_mim]Cl. As a consequence, COSMO-RS delivers quite good estimates for the *S*_*ij*_^∞^ values of these binary mixtures in the [C_4_mim]Cl/[C_12_mim]Cl equimolar mixture, which is confirmed by the low global ARD observed between the experimental and predicted data (15.6%). COSMO-RS describes better the *S*_*ij*_^∞^ in the IL mixture (ARD = 32.3%) than in pure [C_8_mim]Cl (ARD = 49.2%), while the opposite behavior is observed for the capacity values, where ARDs of 12.0 and 15.6% were obtained for [C_8_mim]Cl and IL mixtures, respectively.

In the azeotropic mixtures, the estimated separation factors obtained from both TZVP and TZVPD-FINE are generally lower than the experimental values, though the predictions with TZVP capture better the *S*_*ij*_^∞^/*k*_*j*_^∞^ versus IL composition profiles. At this level of parametrization, the predicted selectivities and capacities usually present systematic variations with the IL mixture composition, with the estimated value often corresponding to a third or half of the observed data, offering a qualitative picture of the curves, which is reasonable considering the model’s fully predictive nature. Herein, the differences between the experimental *S*_*ij*_^∞^ obtained in the IL equimolar mixture and in pure [C_8_mim]Cl are considerably lower than the differences registered for the removal of contaminants from aliphatic hydrocarbons.

COSMO-RS performance in describing the *S*_*ij*_^∞^ and *k*_*j*_^∞^ of specific separations, already evidenced for some pure ionic liquids,^25,26,32,58,59^ is demonstrated here for the first time for imidazolium chloride-based IL mixtures. An evident advantage to use IL mixtures is the melting point depression, which broadens the temperature range in the liquid state. More importantly, the possibility to define specific separation parameters by adjusting the relative proportions of the two (or more) ILs is a feature to be intensively explored, not only addressing the size of the cations but also surely combining anions of very different polarity, increasing the chances to find mixtures matching very specific objectives. The results from this work reveal that COSMO-RS is a powerful tool for an a priori screening of potential separation agents for some industrial relevant separation problems.

#### Relative Volatilities

In azeotropic and extractive distillations, where a solvent/entrainer is added to increase the separation efficiency, the relative volatility is the preferable parameter since it considers data from the liquid and vapor phases.^60^ At infinite dilution, the relative volatility reflects the maximum impact that an entrainer presents over a target mixture, when much lower quantities of the mixture components (solutes) are present in comparison with the amount of the entrainer (solvent).^61^

**Figure 6:**
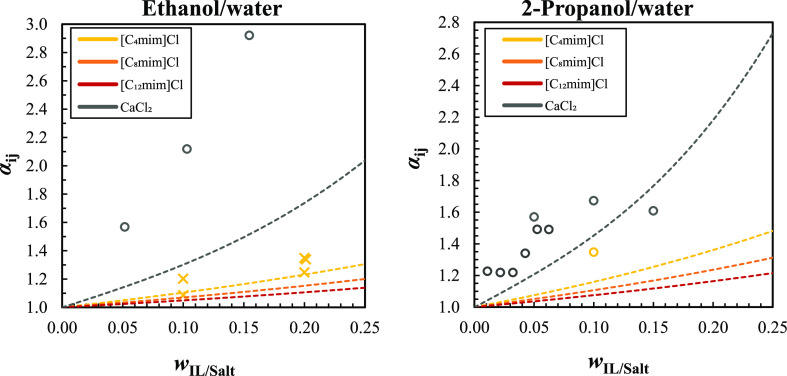
Overview of available experimental and predicted α_*ij*_ (at 101.3 ± 2 kPa) for ethanol/water and 2-propanol/water mixtures in the presence of imidazolium chloride IL and calcium chloride. Open circles (○) and crosses (×) represent the experimental data available for CaCl_2_^68−70^ and [C_4_mim]Cl,^71−74^ respectively, and the dotted lines depict the COSMO-RS predictions using the TZVP parameterization.

The relative volatilities at infinite dilution, α_*ij*_^∞^, for the studied azeotropic mixtures were calculated from the experimental and predicted (with COSMO-RS TZVP) γ_13_^∞^ data and are presented as a function of temperature in Figure S4 and Table S13 of the Supporting Information. The data reveal that α_*ij*_^∞^ decreases as the temperature increases and that COSMO-RS underestimates the experimental values. Additionally, COSMO-RS predicts very similar values for the equimolar mixture and its analogue [C_8_mim]Cl. Experimental α_*ij*_^∞^ are far superior to 1, with values varying between 5 and 14 for ethanol/water, 6–21 for 2-propanol-water, and 27–71 for acetone/methanol. For alcohol/water mixtures, the α_*ij*_^∞^ values suggest that [C_4_mim]Cl would be more effective to break the azeotrope in comparison with the other studied chloride ILs, while [C_8_mim]Cl delivers the best results for acetone/methanol. In real cases, the solvent or entrainer is fed to a distillation process at moderate ratios, often lower than those of the target compounds, and therefore, the behavior might be considerably distinct from the scenario where infinite dilution is assumed. Considering this, the relative volatilities, α_*ij*_ (as defined in eq S12), of the azeotropic mixtures containing different mass fractions of the imidazolium chloride IL were predicted using COSMO-RS (TZVP), and the results are presented in Figure 6 (ethanol/water and 2-propanol/water) and Figure S5 of the Supporting Information (acetone/methanol) and listed in Table S14. Whenever available, the experimental α_*ij*_ were added. Predictions were carried out with the azeotropic composition free of IL/salt.^62−64^ For comparison purposes, the relative volatilities in the presence of calcium chloride, a well-known entrainer to break alcohol/water azeotropes,^65−67^ were estimated and included.

Relative volatilities increase with the addition of entrainers, being the best performance registered for CaCl_2_, followed by [C_4_mim]Cl, [C_8_mim]Cl, and [C_12_mim]Cl. COSMO-RS can capture the α_*ij*_ trends with the salt/IL concentration, delivering lower relative volatilities when compared with the available experimental data points. For the ethanol/water mixture, the model provides a very good description of the α_*ij*_ with [C_4_mim]Cl (ARD of 6%), while higher deviations are registered for CaCl_2_ (ARD 38%). Regarding 2-propanol/water, an ARD of around 14% was obtained for CaCl_2_ and [C_4_mim]Cl, and a deviation of 4% was found for the acetone/methanol mixture with 5% of CaCl_2_. The predicted α_*ij*_ for [C_8_mim]Cl and [C_4_mim]Cl/[C_12_mim]Cl equimolar mixture are very close, and therefore, the latter were omitted.

The patterns suggested by the experimental α_*ij*_^∞^ and predicted α_*ij*_ for alcohol/water azeotropes with pure chloride IL are similar, though the values at infinite dilution are much higher. In both scenarios, the best results were found for [C_4_mim]Cl, followed by [C_8_mim]Cl and [C_12_mim]Cl. Overall, the addition of calcium chloride is clearly more effective to increase the relative volatility of the studied azeotropic mixtures. However, one must take into account that the presence of chloride salts, such as CaCl_2_, NaCl, and KCl, in the solutions might generate precipitation, corrosion, and obstruction issues, thereby increasing costs.^75^ On the other hand, ILs present the great advantage of their physical state and are generally much less corrosive than inorganic salts.^76^ In fact, there is strong evidence that IL, including those with imidazolium cations, act as corrosion inhibitors in metal structures.^76,77^ In this context, the imidazolium chloride ionic liquids, particularly [C_4_mim]Cl, could be good alternatives to break the azeotropic mixtures addressed in this work.

## Conclusions

Aiming to further explore neoteric solvents for industrial applications, imidazolium chloride-based ionic liquids were investigated as potential entrainers for problematic separation problems. Overall, [C_4_mim]Cl is the most promising option to be exploited in the removal of aromatics from aliphatic hydrocarbons, the removal of contaminants from fuels, and the separation of azeotropic mixtures. The predictive COSMO-RS model was employed to further investigate the topic, being able to capture selectivity and capacity trends and offering a fully predictive qualitative picture of these parameters. The model was also applied to predict the relative volatilities of the azeotropes in the presence of the studied ILs, offering a broad perspective of the effects of the entrainers over the target mixtures. It has been shown that the flexibility gained when using IL mixtures as separating agents; combining IL in different proportions, the separation factors can be adjusted for a given separation problem. This work calls for further research in the area, namely, tailoring ionic liquid mixtures for target separation processes by applying the IL designer solvent character.

## Supporting Information Available

General information about the studied ionic liquids and solutes; procedure for column packing and chromatographic experiments; detailed thermodynamic framework and tabled results of activity coefficient at infinite dilution, gas–liquid partition coefficients limiting partial molar excess properties and separation factors; procedure for the estimation of the density data of [C_12_mim]Cl and [C_4_mim]Cl/[C_12_mim]Cl equimolar mixture; selectivities and capacities predicted with COSMO-RS with the TZVP and TZVPD-FINE parametrization levels; and experimental and predicted α_*ij*_ and α_*ij*_^∞^ for the selected azeotropic mixtures with the chloride-based entrainers (PDF)
