# Supplementary material for: Imidazolium Chloride Ionic Liquid Mixtures as Separating Agents: Fuel Processing and Azeotrope Breaking
Source: Energy Fuels. 2022 Jul 26;36(15):8552–61. doi: 10.1021/acs.energyfuels.2c01724 (PMC9778084; doi:10.1021/acs.energyfuels.2c01724)
Supplement: Supplementary file 1 — ef2c01724_si_001.pdf [file ef2c01724_si_001.pdf]

Supporting Information

Imidazolium chloride ionic liquid mixtures as separating agents: fuel processing and azeotrope breaking

Sérgio M. Vilas-Boas[a,b], Mónia A. R. Martins[b], Fábio R. Tentor[a,c], Gabriel Teixeira[a,b], Juliana G. Sgorlon[c], João A. P. Coutinho[b], Olga Ferreira[a], and Simão P. Pinho[a*]

[a]Centro de Investigação de Montanha (CIMO), Instituto Politécnico de Bragança, Campus de Santa Apolónia, 5300-253 Bragança, Portugal

[b]CICECO – Aveiro Institute of Materials, Department of Chemistry, University of Aveiro, 3810-193 Aveiro, Portugal

[c]Federal University of Technology of Paraná – UTFPR, Rua Marcílio Dias, 635, 86812-460, Apucarana, Parana, Brazil

*Corresponding author: Simão P. Pinho

e-mail address: spinho@ipb.pt

Phone: +351 273303086

Fax: +351 273313051

Section S1 – Experimental details

Table S1. Chemical structure of the cation, name, molar mass, melting temperature and purity (mass fraction) of the studied imidazolium-chloride ionic liquids acquired from Iolitec.

**Chemical Formula Cation**	**Chemical name and abbreviation**	**Molar mass (g/mol)**	**Melting temperature (K)**	**Mass fraction purity[d]**
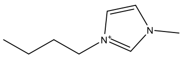	1-butyl-3-methylimidazolium chloride – $[C_4mim]Cl$	174.671	341.95 [1,a]	≥ 0.99
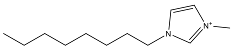	1-octyl-3-methylimidazolium chloride – $[C_8mim]Cl$	230.777	285.41 [1,b]	≥ 0.99
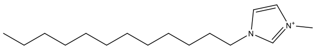	1-dodecyl-3-methylimidazolium chloride – $[C_{12}mim]Cl$	286.884	424.9 [1,c]	≥ 0.98

[a]The IL presents a glass transition at 197.35 K.[1]

[b]The IL presents a glass transition at 210.85 K.[1]

[c]The IL presents a liquid crystal transition at 317.65 K.[1]

[d]Provided by the supplier (Iolitec).

Table S2. Chemical structure, supplier, normal boiling temperature and purity of solutes.

Family	Compound	Chemical structure	Supplier	Boiling temperature (K)[b]	Purity (mass fraction)
	water	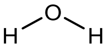	- [a]	373.15	- [a]
Alkanes	heptane	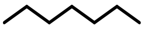	Carlo Erba	371.53	≥ 0.990
	octane	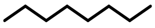	Aldrich	398.77	≥ 0.990
	nonane	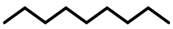	Aldrich	423.91	≥ 0.990
	decane	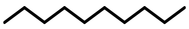	Aldrich	447.20	≥ 0.990
Cycloalkanes	cyclohexane	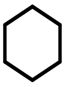	Aldrich	353.90	≥ 0.990
	methylcyclohexane	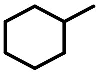	Aldrich	374.00	≥ 0.990
Ketones	propanone (acetone)	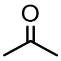	Aldrich	329.30	≥ 0.999
	2-butanone	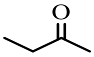	Aldrich	353.00	≥ 0.990
Ethers	ethoxyethane (diethyl ether)	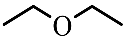	Aldrich	307.70	≥ 0.999
Cyclic Ethers	oxolane (THF)	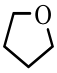	Aldrich	339.00	≥ 0.999
	1,4-dioxane	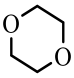	Aldrich	374.30	≥ 0.998
Aromatic Hydrocarbons	benzene	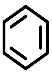	Aldrich	353.22	≥ 0.998
	toluene	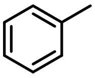	Aldrich	383.75	≥ 0.998
	ethylbenzene	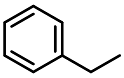	Aldrich	409.35	≥ 0.998
	p-xylene	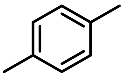	Aldrich	411.51	≥ 0.990
Esters	methyl acetate	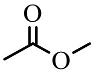	Aldrich	330.00	≥ 0.998
	vinyl acetate	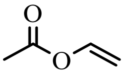	Riedel-de-Häen	345.70	≥ 0.990

	ethyl acetate	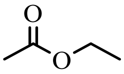	Aldrich	350.20	≥ 0.998
Alcohols	methanol	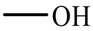	Aldrich	337.80	≥ 0.999
	ethanol	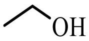	Aldrich	351.50	≥ 0.998
	1-propanol	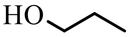	Aldrich	370.30	≥ 0.999
	2-propanol	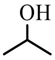	Fluka	355.50	≥ 0.999
	2-methyl-1-propanol (isobutanol)	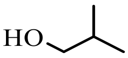	Aldrich	380.80	≥ 0.995
	1-butanol	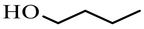	Aldrich	390.60	≥ 0.998
	2-butanol	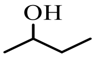	Aldrich	372.00	≥ 0.995
	2-methyl-2-propanol (*tert*-butanol)	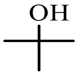	Aldrich	355.50	≥ 0.997
	acetonitrile	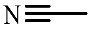	Fluka	355.15	≥ 0.999
	pyridine	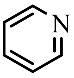	Aldrich	388.15	≥ 0.998
	thiophene	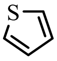	Aldrich	357.15	≥ 0.990

[a]Ultrapure water (resistivity of 18.2 MΩ·cm, free particles ≥ 0.22 μm and total organic carbon < 5 $\mu g \cdot dm^{-3}$) was used in all the experiments involving this solute.

[b]The boiling temperature was obtained from Yaws.[2]

Procedure for column packing and chromatographic experiments

An all-glass column (1 m length and 4 mm internal diameter) was filled with the stationary phase composed by a mixture of 45-55% (in mass) of the IL and the solid support (Chromosorb W/AW – DMCS, Grace, 100–120 mesh). The mixture of [C_4mim]Cl and [C_{12}mim]Cl was prepared in the mole ratio of 0.503:0.497 ([C_4mim]Cl:[C_{12}mim]Cl) by weighting 1.7181 g of [C_4mim]Cl and 2.7875 of [C_{12}mim]Cl in a dry-argon glove-box using an analytical balance model ALS 220-4 N from Kern (repeatability of 0.2 mg). To achieve a uniform coating, methanol was used to dissolve the (IL or IL mixture + Chromosorb) mixture under continuous stirring, followed by its evaporation under vacuum until the deviations between the final and original masses of the mixture were lower than 0.1 mg. Then, the column was placed into a Varian CP-3380 gas chromatograph (GC) coupled with a thermal conductivity detector (TCD) and an on-column injector. To perform the experiments, helium was used as the carrier gas, and temperatures of the injector and detector were kept at 473.15 K and 523.15 K, respectively. Before the injections, the columns were pre-conditioned at 393.15 K with helium for at least 6 h to remove possible contaminants left.

During the experiments, a precision gas flowmeter (Agilent, model 5067-0223) was used to measure the exit flow rate (relative uncertainty of 6%), the outlet temperature (±0.1 K) and the atmospheric pressure (±0.05 atm). Besides, the column inlet pressure was registered by a Swagelok S model pressure transducer with an accuracy of 0.25% BFSL. To ensure the solutes were in the infinite dilution state, samples of around 0.2 μl were injected along with air, the non-retained component. For each injection, the retention times were calculated by the difference between the retention time of the solute, t_r, and the retention time of air, t_g. For each solute-IL system, at least five data points were obtained in the temperature range between (373.15-423.15) K for alcohols and water, and (333.15-383.15) K for all other organic solutes. To check the reproducibility of the results, at least two independent injections were performed for all the solutes at each temperature. Furthermore, the activity coefficient measurements of ten representative solutes (including compounds from different families) were carried out in two independent columns, with different packing percentages, resulting in a global coefficient of variation of 3.8%.

Section S2 - Thermodynamic framework

Activity coefficient at infinite dilution

When an infinitesimal amount of a solute (1) is introduced into a column with the carrier gas (2) and the nonvolatile stationary phase (3), the activity coefficient at infinite dilution of the solute γ_{13}^{∞} *can* be calculated using the following equation:[3,4]

$$\ln \gamma_{13}^{\infty} = \ln\left(\frac{n_3 RT}{V_N p_1^*}\right) - \frac{p_1^*(B_{11} - V_1^*)}{RT} + \frac{p_0 J_2^3(2B_{12} - V_1^{\infty})}{RT} \qquad \textbf{(S1)}$$

where n_3 is the number of moles of the ionic liquid packed in the column, R is the ideal gas constant, T is the column absolute temperature (controlled by the GC oven), V_N is the net retention volume of the solute, p_1^* is the saturated vapor pressure of the solute at the column temperature, B_{11} is the second virial coefficient of the pure solute, V_1^* is the molar volume of the pure solute, p_0 is the outlet column pressure, J_2^3 is the pressure correction term, B_{12} is the second virial coefficient of the solute in the carrier gas, and V_1^{∞} is the partial molar volume of the solute at infinite dilution.

All the thermophysical properties of the pure solute required in **Eq. (S1)**, namely the vapor pressure, the molar volume, and the second virial coefficient, were retrieved from the DIPPR 801 database.[5] The mixed second virial coefficient of the solute and the carrier gas (B_{12}) were estimated by the approach proposed by Tsnonopoulos,[6] and discussed in detail by Poling et al.[7] The net retention volume (V_N), representing the total volume of solute that passes through the column, is obtained by the following relationship:

$$V_N = (J_2^3)^{-1} U_0 (t_r - t_g) \qquad \textbf{(S2)}$$

in which U_0 is the outlet column volumetric flow rate, and t_r and t_g are the retention times of the solute and the non-retained substance introduced into the column along with the solute (frequently air), respectively. Since the flow rate is registered after the carrier gas passes though the detector, the following correction is required to retrieve the flow rate at the column conditions:

$$U_0 = U_f \frac{p_f}{p_0} \cdot \frac{T}{T_f} \quad \textbf{(S3)}$$

where U_f, p_f and T_f are the volumetric flow, the pressure, and the temperature measured by the flowmeter after the carrier gas passes through the detector, respectively. The pressure correction factor, J_2^3, required in **Eq. (S1)** and **Eq. (S2)** to compensate the pressure drop in the column, is expressed as:[3,8]

$$J_2^3 = \frac{2\left(\frac{p_i}{p_0}\right)^3 - 1}{3\left(\frac{p_i}{p_0}\right)^2 - 1} \quad \textbf{(S4)}$$

where p_i is the inlet pressure of the column.

Whenever the γ_{13}^{∞} are available at different temperatures, the data can be used to derive some excess partial molar properties, namely the enthalpy ($\bar{H}_m^E$), entropy ($\bar{S}_m^E$) and, Gibbs energy ($\bar{G}_m^E$) by the following equations:

$$\bar{H}_m^E = R \cdot \left(\frac{\partial ln\gamma_{13}^{\infty}}{\partial \left(\frac{1}{T}\right)} \right)_{p,x} \quad \textbf{(S5)}$$

$$\bar{G}_m^{E,\infty} = RT \ln \gamma_{13}^{\infty} \quad \textbf{(S6)}$$

$$\bar{S}_m^E = \frac{\bar{H}_m^E - \bar{G}_m^{E,\infty}}{T} \quad \textbf{(S7)}$$

in which the subscripts p and x indicate constant pressure and constant composition conditions, respectively.

Gas-liquid partition coefficients

The gas-liquid partition coefficient of solute partitioning between the stationary phase (IL) and the gas phase (carrier gas) can also be calculated form the GC retention times using the following equation:

$$ln(K_L) = \frac{c_1^3}{c_1^2} = ln\left(\frac{V_N \rho_3}{m_3}\right) - \frac{P_0 J_2^3 (2B_{12} - V_1^\infty)}{RT} \quad \textbf{(S8)}$$

in which c is the molar concentration of the solute, ρ_3 is the density of the IL and m_3 is the mass of the IL packed into the column.

Separation factors

The selectivities, S_{ij}^∞, and capacities, k_j^∞, are useful parameters to assess the suitability of the ionic liquid for a specific separation problem, and are directly related with the γ_{13}^∞ by:

$$S_{ij}^\infty = \frac{\gamma_{i3}^\infty}{\gamma_{j3}^\infty} \quad \textbf{(S9)}$$

$$k_j^\infty = \frac{1}{\gamma_{j3}^\infty} \quad \textbf{(S10)}$$

where the subscripts i and j represent the target solutes, being j the solute with the lower activity coefficient value for a given separation, and 3 refers to the ionic liquid. For the selection of an appropriate separation agent, high selectivities and high capacities are desirable, though these values often present an inverse relationship for several specific separation problems.[9] To best assess the suitability of the solvent by a single quantity, the solvent performance index is included:[10–12]

$$\mathrm{Q}_{ij}^\infty = S_{ij}^\infty k_j^\infty \quad \textbf{(S11)}$$

While S_{ij}^∞, k_j^∞, and Q_{ij}^∞ are very useful parameters to evaluate potential separation agents for liquid-liquid extraction processes, the relative volatility (α_{ij}) is often desirable for distillation process. It is directly obtained from vapor-liquid equilibrium data:[10,13,14]

$$\alpha_{ij} = \frac{y_i/x_i}{y_j/x_j} = \frac{\gamma_i p_i^0}{\gamma_j p_j^0} \quad \textbf{(S12)}$$

where y and x are the molar compositions of the vapor and liquid phases, respectively, γ is the activity coefficient, p^0 is the pure compound vapor pressure, and the subscripts i and j stand for the targets components to be fractionated, being j the less volatile compound.

Eq. **(S12)** might also be applied when a solvent/entrainer is added in the target mixture if the compositions in the vapor and liquid phases, or the solute's activity coefficient and vapor pressures, are known. Additionally, a relative volatility at infinite dilution can be also be defined as:[15]

$$\alpha_{ij}^{\infty} = \frac{\gamma_{i3}^{\infty} p_i^0}{\gamma_{j3}^{\infty} p_j^0} \quad \textbf{(S13)}$$

where 3 stands for the entrainer/solvent, and the subscripts *i* and *j* represent the solutes at infinite dilution.

Statistical analysis

The deviations between the experimental and predicted separation factors (e.g., S_{ij}^{∞}, k_j^{∞}, Q_{ij}^{∞}, α_{ij}) were assessed by the average relative deviation (ARD), calculated as follows:

$$\text{ARD (\%)} = \frac{1}{n}\sum_i \left(\frac{SF_i^{\text{exp}} - SF_i^{\text{calc}}}{SF_i^{\text{exp}}}\right) * 100 \quad \textbf{(S14)}$$

where SF stands for the analyzed separation factor, the superscripts "exp" and “calc" mean the experimental and calculated, respectively, n is the total number of data points and i covers all the separation set under study.

Section S3 – Results and discussion

Activity coefficients at infinite dilution

Table S3. Activity coefficients at infinite dilution of the organic compounds and water in [C_8mim]Cl, [C_4mim]Cl/[C_{12}mim]Cl equimolar mixture, and [C_{12}mim]Cl.[a]

Solutes		**[C_8mim]Cl[b]**						**[C_4mim]Cl /[C_{12}mim]Cl equimolar mixture[c]**						**[C_{12}mim]Cl[d]**					
	T/K	333.15	343.15	353.15	363.15	373.15	383.15	333.15	343.15	353.15	363.15	373.15	383.15	333.15	343.15	353.15	363.15	373.15	383.15
heptane		72.468	65.034	57.437	54.628	51.097	47.199	30.188	28.366	27.190	25.650	24.679	23.763	5.784	5.821	5.911	6.017	6.100	6.219
octane		57.907	53.720	50.495	47.170	44.635	42.815	26.017	24.916	24.099	-	23.011	22.606	5.961	5.973	6.144	6.119	6.279	6.238
nonane		48.259	46.250	44.375	42.823	41.727	40.552	24.134	23.821	23.523	23.225	22.933	22.487	6.417	6.529	6.714	6.767	6.965	7.032
decane		49.640	46.968	45.499	44.258	43.692	42.743	25.324	-	24.817	24.461	24.177	23.807	7.355	7.382	7.490	7.521	7.545	7.698
cyclohexane		41.088	34.909	30.205	26.477	23.569	20.473	20.597	17.415	15.522	13.793	12.602	11.858	3.973	3.809	3.802	3.624	3.665	3.558
methylcyclohexane		41.251	35.602	31.291	27.869	25.423	22.935	19.542	17.252	15.533	14.472	13.565	12.912	4.099	4.059	4.028	4.044	4.020	3.977
benzene		2.075	2.075	2.074	2.073	2.074	2.074	1.992	1.976	1.960	1.950	1.943	1.925	1.214	1.239	1.261	1.281	1.302	1.318
toluene		2.705	2.746	2.773	2.820	2.850	2.893	2.611	2.624	2.643	2.638	2.658	2.648	1.467	1.504	1.556	1.586	1.672	1.695
ethylbenzene		3.975	4.029	4.067	4.112	4.145	4.186	3.501	3.490	3.505	3.505	3.511	3.513	1.840	1.903	1.936	2.000	2.069	2.096
p-xylene		3.858	3.929	3.970	3.959	3.964	4.032	3.554	3.564	3.596	3.597	3.616	3.622	1.875	1.942	1.988	2.037	2.128	2.161
methyl acetate		3.651	3.465	3.296	3.156	3.076	2.976	3.370	3.208	3.064	2.984	2.897	2.852	2.496	2.448	2.397	2.350	2.315	2.275
ethyl acetate		4.922	4.698	4.558	4.418	4.293	4.209	4.589	4.346	4.196	4.080	3.990	3.820	2.919	2.866	2.851	2.800	2.777	2.772
vinyl acetate		3.419	3.336	3.256	3.190	3.120	3.083	3.333	3.213	3.123	3.059	3.032	2.974	2.463	2.432	2.397	2.395	2.353	2.331
THF		3.112	2.987	2.857	2.761	2.684	2.623	2.836	2.667	2.603	2.516	2.423	2.393	1.668	1.637	1.625	1.588	1.613	1.556
1,4-dioxane		2.120	2.068	2.040	2.032	2.008	2.002	2.089	2.050	2.031	1.999	1.971	1.934	1.670	1.660	1.644	1.630	1.639	1.645
diethyl ether		24.672	21.780	19.703	17.790	16.533	15.458	15.023	13.623	12.168	11.157	10.250	9.900	4.797	4.646	4.481	4.319	4.106	4.072
acetonitrile		0.963	0.961	0.960	0.959	0.955	0.953	0.977	0.974	0.977	0.974	0.974	0.971	1.174	1.159	1.151	1.138	1.118	1.100
pyridine		0.973	0.982	0.992	0.999	1.009	1.018	1.123	1.120	1.118	1.109	1.105	1.104	1.033	1.037	1.039	1.042	1.043	-
thiophene		1.080	1.108	1.136	1.158	1.186	1.227	1.086	1.104	1.126	1.140	1.161	1.181	0.808	0.834	0.857	0.881	0.916	-
acetone		2.555	2.434	2.312	2.218	2.139	2.086	2.443	2.303	2.214	2.148	2.072	1.992	2.067	1.989	1.941	1.897	1.856	1.813
2-butanone		2.739	2.670	2.612	2.565	2.525	2.508	2.739	2.634	2.553	2.507	2.454	2.431	2.113	2.093	2.058	2.043	2.029	2.021
**Alcohols/water**	T/K	373.15	383.15	393.15	403.15	413.15	423.15	373.15	383.15	393.15	403.15	413.15	423.15	373.15	383.15	393.15	403.15	413.15	423.15
methanol		0.086	0.090	0.096	0.101	0.106	0.114	0.089	0.093	0.097	0.101	0.105	0.111	0.089	0.091	0.095	0.098	0.103	0.106
ethanol		0.135	0.141	0.149	0.155	0.163	0.170	0.140	0.145	0.151	0.158	0.164	0.173	0.129	0.134	0.137	0.142	0.146	0.152
1-propanol		0.150	0.158	0.169	0.179	0.190	0.202	0.155	0.163	0.172	0.180	0.191	0.203	0.130	0.142	0.140	0.148	0.157	0.168
2-propanol		0.199	0.212	0.224	0.237	0.252	0.270	0.204	0.214	0.225	0.236	0.249	0.267	0.171	0.176	0.183	0.195	0.207	0.217
isobutanol		0.162	0.174	0.185	0.196	0.209	0.224	0.168	0.176	0.186	0.196	0.209	0.220	0.131	0.139	0.147	0.156	0.164	0.175
1-butanol		0.170	0.179	0.191	0.202	0.215	0.228	0.175	0.185	0.195	0.205	0.217	0.232	0.135	0.145	0.150	0.159	0.169	0.181
2-butanol		0.222	0.237	0.259	0.280	0.298	0.324	0.226	0.241	0.257	0.273	0.293	0.320	0.179	0.191	0.202	0.215	0.228	0.245
tert-butanol		0.315	0.340	0.374	0.403	0.438	0.479	0.290	0.312	0.333	0.356	0.393	0.425	0.232	0.252	0.255	0.274	0.297	0.320
water		0.055	0.060	0.067	0.073	0.079	0.085	0.057	0.062	0.068	0.073	0.080	0.088	0.055	0.060	0.065	0.069	0.076	0.089

[a]The estimated uncertainties in pressure, temperature and γ_{13}^{∞} are: $u(T)$ = 0.1 K, $u_r(p)$ = 0.05 and $u(\gamma_{13}^{\infty})$ = 0.04. [b]Colunm Packing: 52.7% of IL, $n_3 = 11.31$ mmol; [c]Colunm Packing: 46.5% of IL, $n_3 = 9.35$ mmol; [d]Colunm Packing: 46.4% of IL, $n_3 = 7.38$ mmol.

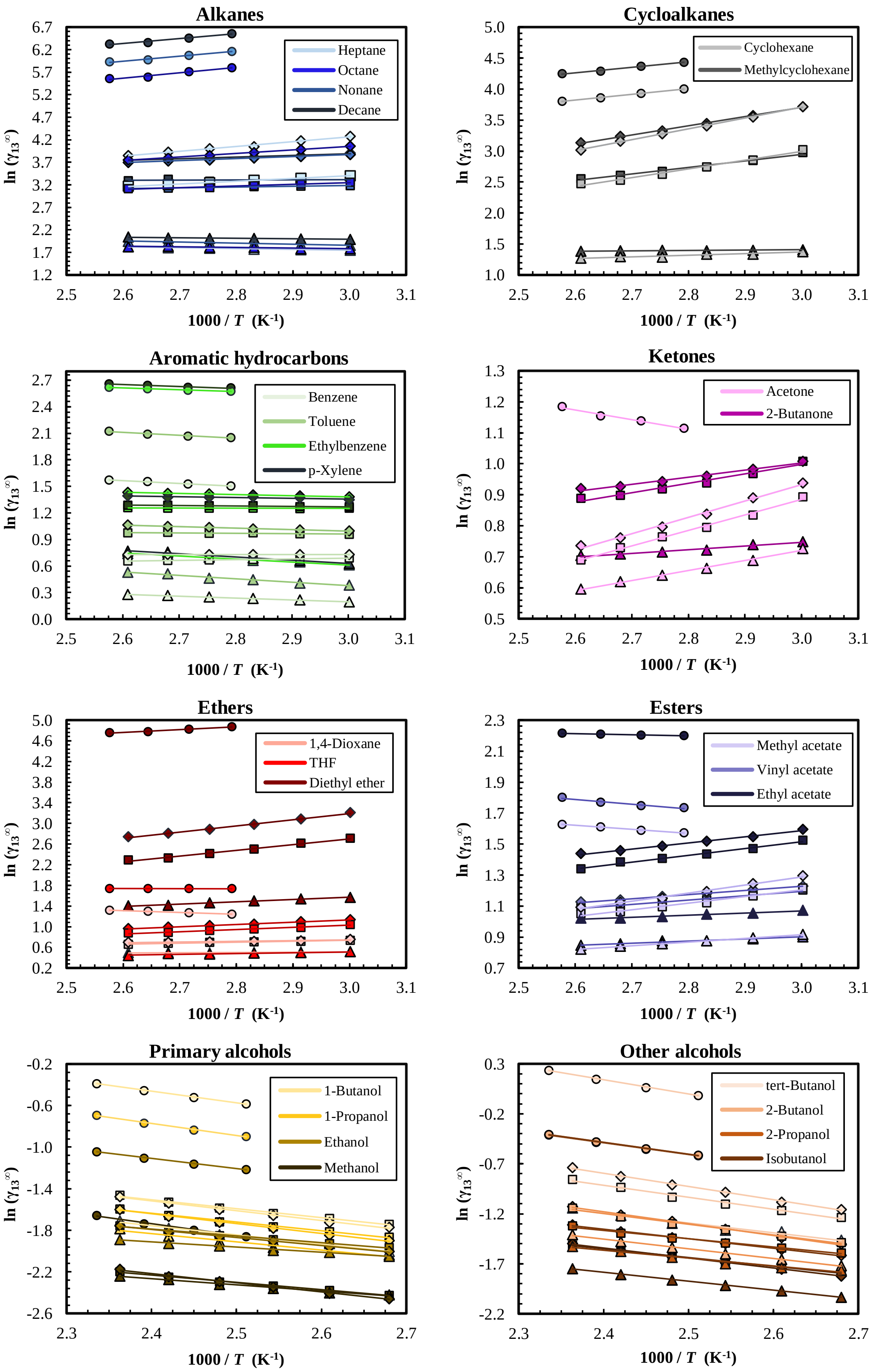
Alkanes
Heptane
Octane
Nonane
Decane
ln (γ_{13}^{∞})
1000 / T (K^{-1})
Cycloalkanes
Cyclohexane
Methylcyclohexane
ln (γ_{13}^{∞})
1000 / T (K^{-1})
Aromatic hydrocarbons
Benzene
Toluene
Ethylbenzene
p-Xylene
ln (γ_{13}^{∞})
1000 / T (K^{-1})
Ketones
Acetone
2-Butanone
ln (γ_{13}^{∞})
1000 / T (K^{-1})
Ethers
1,4-Dioxane
THF
Diethyl ether
ln (γ_{13}^{∞})
1000 / T (K^{-1})
Esters
Methyl acetate
Vinyl acetate
Ethyl acetate
ln (γ_{13}^{∞})
1000 / T (K^{-1})
Primary alcohols
1-Butanol
1-Propanol
Ethanol
Methanol
ln (γ_{13}^{∞})
1000 / T (K^{-1})
Other alcohols
tert-Butanol
2-Butanol
2-Propanol
Isobutanol
ln (γ_{13}^{∞})
1000 / T (K^{-1})

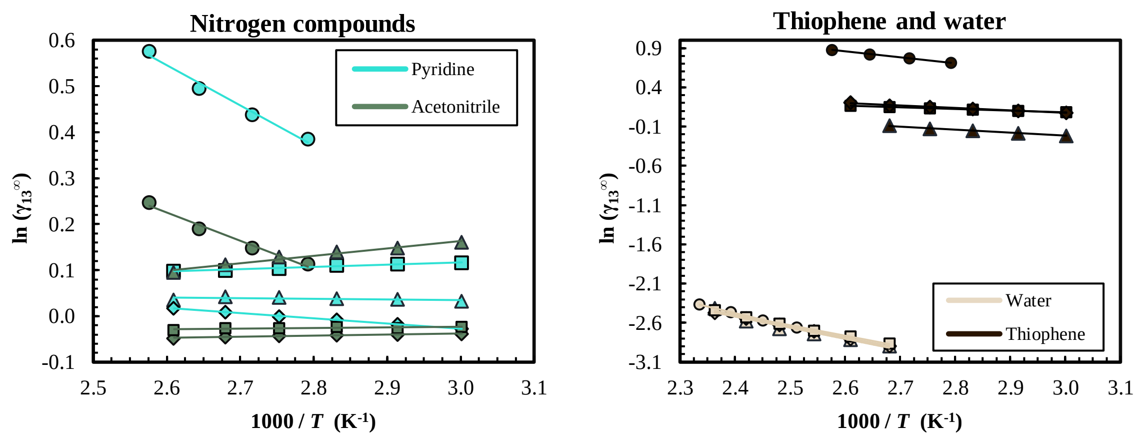

Figure S1. Representation of $\ln(\gamma_{13}^{\infty})$ as function of 1000/T of the investigated solutes in: [C_4mim]Cl (●) – from literature[16], [C_8mim]Cl (◆), [C_4mim]Cl/[C_{12}mim]Cl equimolar mixture (■), and [C_{12}mim]Cl (▲).

Gas-liquid partition coefficients

Table S4. Gas-liquid partition coefficients of water and several organic compounds in [C_8mim]Cl, [C_4mim]Cl/[C_{12}mim]Cl equimolar mixture, and [C_{12}mim]Cl.[a]

Solutes	[C_8mim]Cl[b]						[C_4mim]Cl /[C_{12}mim]Cl equimolar mixture[c]						[C_{12}mim]Cl[d]					
T/K	333.15	343.15	353.15	363.15	373.15	383.15	333.15	343.15	353.15	363.15	373.15	383.15	333.15	343.15	353.15	363.15	373.15	383.15
heptane	6.019	4.783	3.961	3.116	2.545	2.146	14.443	10.964	8.367	6.657	5.272	4.264	58.034	41.107	29.594	21.746	16.383	12.507
octane	19.847	14.538	10.813	8.304	6.446	5.043	44.161	31.340	22.656	-	12.507	9.556	148.374	100.584	68.339	49.205	35.203	26.582
nonane	62.233	42.002	29.237	20.831	15.092	11.231	124.402	81.538	55.155	38.415	27.470	20.283	360.190	228.900	148.607	101.326	69.469	49.746
decane	158.784	103.442	68.167	46.203	31.755	22.623	312.449	-	123.228	83.644	55.261	39.579	824.804	506.274	318.420	208.956	141.298	96.476
cyclohexane	5.730	4.974	4.335	3.805	3.349	3.073	11.426	9.969	8.436	7.305	6.266	5.308	45.604	35.076	26.481	21.366	16.548	13.581
methylcyclohexane	10.772	8.977	7.528	6.369	5.367	4.657	22.731	18.523	15.165	12.267	10.062	8.276	83.426	60.579	44.971	33.736	26.081	20.626
benzene	112.358	82.242	61.616	47.140	36.732	29.144	116.980	87.405	65.213	50.710	39.194	31.432	147.817	105.974	77.928	58.655	44.970	35.207
toluene	240.836	166.404	118.530	85.785	63.800	48.151	249.493	174.126	124.381	91.712	68.429	52.635	341.747	233.742	162.417	117.211	83.544	63.135
ethylbenzene	406.700	269.684	184.753	129.646	93.436	68.689	461.645	311.238	214.360	152.141	110.369	81.884	676.279	439.163	298.366	204.850	143.841	105.347
p-xylene	453.898	298.837	203.995	144.806	104.807	76.292	492.488	329.332	225.228	159.393	114.938	84.980	718.578	465.150	313.237	216.366	150.545	109.353
methyl acetate	30.476	23.931	19.179	15.598	12.703	10.604	33.008	25.842	20.635	16.499	13.492	11.068	34.310	26.061	20.284	16.100	12.966	10.654
ethyl acetate	44.968	33.954	25.863	20.182	16.043	12.882	48.215	36.693	28.093	21.857	17.268	14.203	58.346	42.807	31.799	24.475	19.056	15.023
vinyl acetate	55.355	41.195	31.395	24.363	19.331	15.469	56.766	42.766	32.733	25.412	19.903	16.042	59.124	43.466	32.796	24.948	19.699	15.715
THF	47.204	36.584	29.073	23.334	18.960	15.577	51.780	40.970	25.615	31.919	21.004	17.086	67.783	51.344	39.305	31.176	24.242	20.173
1,4-dioxane	236.325	170.498	124.580	92.184	70.143	53.933	239.728	171.940	125.113	93.702	71.514	55.861	230.832	163.396	118.831	87.833	66.029	50.409
diethyl ether	2.140	2.002	1.766	1.588	1.413	1.261	3.703	3.199	2.860	2.533	2.275	1.970	8.927	7.218	5.971	5.033	4.361	3.677
acetonitrile	265.292	194.817	146.080	111.631	87.075	68.934	261.311	192.187	143.584	109.936	85.406	67.749	167.480	124.265	93.710	64.732	72.306	45.885
pyridine	832.969	565.922	394.495	282.873	206.700	154.247	722.004	496.309	350.255	254.887	188.735	142.385	604.106	412.323	289.678	208.508	153.618	-
thiophene	248.111	175.301	127.041	94.642	71.648	54.609	246.614	176.043	128.206	96.165	73.162	56.776	255.204	179.241	129.614	95.655	71.280	-
acetone	42.612	33.575	27.093	22.070	18.195	15.071	44.539	35.479	28.290	22.786	18.784	15.789	40.537	31.604	24.822	19.826	16.109	13.329
2-butanone	85.386	63.630	48.378	37.453	29.484	23.434	85.339	64.487	49.494	38.317	30.346	24.191	85.168	62.447	47.222	36.132	28.193	22.337
**Alcohols/water** T/K	373.15	383.15	393.15	403.15	413.15	423.15	373.15	383.15	393.15	403.15	413.15	423.15	373.15	383.15	393.15	403.15	413.15	423.15
methanol	460.719	332.768	243.042	182.047	139.237	104.908	445.350	323.881	241.115	182.495	139.900	107.622	340.808	254.489	189.048	143.471	109.415	86.327
ethanol	454.159	321.561	229.709	170.176	127.195	127.200	436.363	312.188	226.760	168.115	126.416	95.944	364.986	260.675	192.972	143.006	109.387	83.813
1-propanol	815.826	561.223	387.967	277.289	201.813	148.829	789.755	542.332	381.933	275.648	200.925	148.327	721.266	479.690	359.153	256.359	187.190	137.362
2-propanol	351.309	243.816	174.173	127.143	94.454	70.818	342.197	241.145	173.563	127.841	95.794	71.693	313.629	224.855	163.492	118.839	88.023	67.571
isobutanol	1094.245	722.970	494.515	347.739	249.305	181.579	1057.223	714.620	491.909	347.842	249.353	184.520	1040.631	692.485	476.427	335.882	243.381	178.339
1-butanol	1518.516	1010.695	679.006	470.798	332.088	240.188	1472.406	973.527	665.285	464.816	330.377	237.164	1466.386	953.352	663.932	459.948	324.612	232.090
2-butanol	597.322	405.313	276.558	194.953	142.472	103.928	587.422	399.191	279.042	199.405	144.958	105.285	569.427	386.190	272.516	194.039	142.537	105.518
tert-butanol	228.532	158.257	110.167	80.156	59.025	43.980	248.101	172.731	123.750	90.843	65.823	49.686	238.285	163.968	123.822	90.413	66.659	50.514
water	2351.522	1560.283	1043.073	721.953	508.816	368.743	2282.523	1504.927	1035.880	718.332	506.183	358.983	1807.948	1204.124	825.833	582.386	405.400	267.762

[a]The estimated uncertainties in the pressure, temperature and γ_{13}^{∞} are: $u(T)$ = 0.1 K, $u(p)$ = 0.05 and $u(\gamma_{13}^{\infty})$ = 0.04. [b]Colunm Packing: 52.7% of IL, $n_3 = 11.31$ mmol; [c]Colunm Packing: 46.5% of IL, $n_3 = 9.35$ mmol; [d]Colunm Packing: 46.4% of IL, $n_3 = 7.38$ mmol.

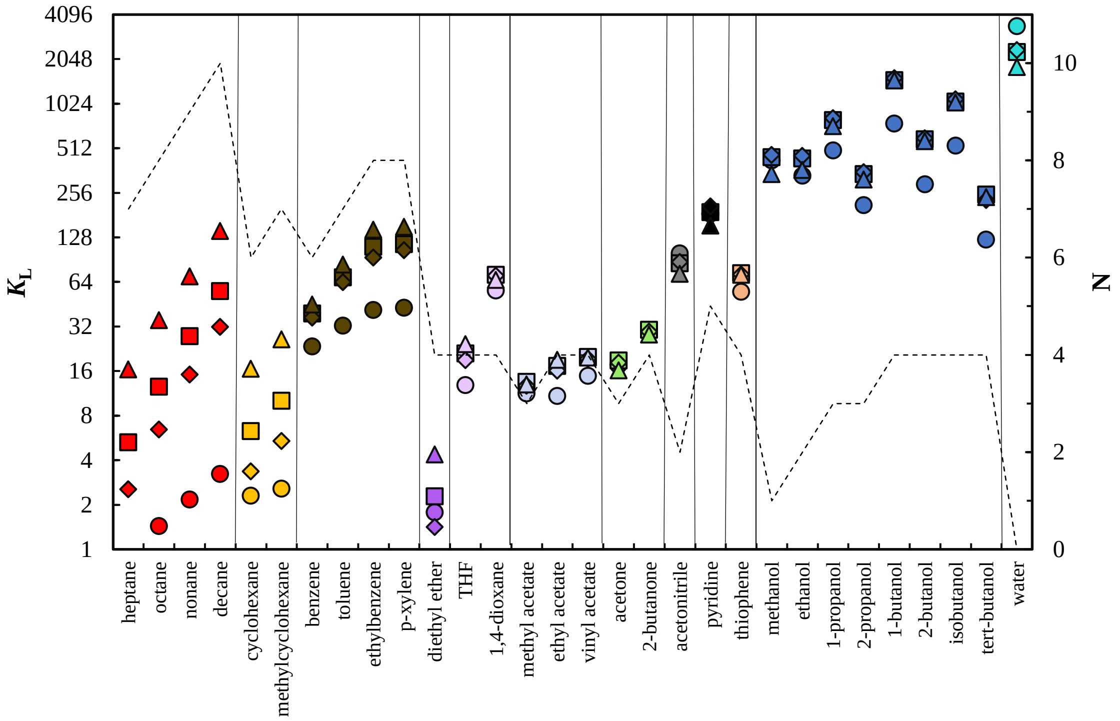

Figure S2. Gas-liquid partition coefficients (K_L) of several organic solutes and water at 373.15 K in: [C$_4$mim]Cl (●) – from literature[16], [C$_8$mim]Cl (◆), [C$_4$mim]Cl/[C$_{12}$mim]Cl equimolar mixture (■), and [C$_{12}$mim]Cl (▲). Dotted line represents the number of carbons in the solute's structure, N.

As can be seen in **Eq. (S8)**, the calculation of K_L depends on the availability of the IL density data, which were experimentally determined before for [C$_8$mim]Cl.[17–25] For this IL, the average literature density data were used to compute the K_L values listed in **Table S4**. Since no experimental density data were found for [C$_{12}$mim]Cl and for [C$_{12}$mim]Cl/[C$_4$mim]Cl equimolar mixture, the approach proposed by Rebelo et al.,[26] and previously employed by us,[27] was used to estimate their densities. Details about the procedure employed is presented in the following subsection.

Estimation of the density data

As discussed in detail by Rebelo and co-authors,[26] the molar volumes (V_m) of a series of [C$_n$mim]-based IL with a common anion follows a linear trend with C$_n$, which represents the size of the alkyl chain attached to the imidazolium ring. Assuming these IL behave as an "ideal" mixture of the cation and anion, their molar volume can be calculated as the sum of the induvial contributions for the cation and anion, where the IL V_m linearly increases as the alkyl cation alkyl chain increases. Therefore, the molar volume of [C$_{12}$mim]Cl, and consequently the densities, might be estimated from the available molar volumes of imidazolium-chloride IL with lower cation alkyl chains. Likewise, the molar volume of the equimolar mixture of [C$_4$mim]Cl and [C$_{12}$mim]Cl can also be predicted assuming the "ideal" behavior.

In this context, a literature review on the available density data for the imidazolium chloride based IL was performed (**Table S5**), and the data used to estimate the densities of [C$_{12}$mim]Cl and [C$_4$mim]Cl/[C$_{12}$mim]Cl between (298.2-363.2) K. The estimated density data are presented in **Table S6** along with the average experimental data for retrieved from literature for [C$_8$mim]Cl, which were used to calculate the gas-liquid partition coefficients presented in **Table S4** .

To check the reliability of the predictions, the density of [C$_8$mim]Cl was also estimated using the available data for [C$_n$mim]Cl IL, with n ranging from 2 to 6, achieving an average relative deviation (from the average experimental data found in literature) of 0.21%. Moreover, this methodology has already been successfully used by us to predict the densities of [C$_{12}$mim][BF$_4$],[27] with an average relative deviation of 0.21% of the experimental data available in literature. To the best of our knowledge, no density data is available for comparison for [C$_{12}$mim]Cl or for the equimolar mixture.

Table S5. Overview of the available density data for chloride imidazolium ionic liquids.

Ionic liquid	Nº data points	Temperature range (K)	Density range (g·cm^{-3})	Reference
**[C$_2$mim]Cl**	11	313.4-364.9	1.124-1.113	[28]
	7	333.1-363.1	1.124-1.107	[20]
**[C$_4$mim]Cl**	14	308.2-373.2	1.079-1.044	[16]
	11	313.1-364.8	1.073-1.045	[28]
	13	303.1-363.1	1.080-1.047	[20]
	6	348.2-373.2	1.054-1.041	[29]
	6	348.2-373.2	1.056-1.043	[30]
	4	298.2-333.2	1.082-1.045	[31]
	5	298.2-328.2	1.074-1.056	[32]
	5	343.2-363.2	1.058-1.047	[33]
	3	298.2-318.2	1.082-1.071	[34]
	5	303.2-323.2	1.080-1.069	[35]
	4	298.2-313.2	1.075-1.063	[36]
**[C$_6$mim]Cl**	13	303.2-363.2	1.035-1.002	[28]
	15	293.2-363.2	1.044-1.004	[20]
	4	298.2-363.2	1.041-1.005	[31]
	7	293.2-353.2	1.043-1.007	[19]
	2	298.2-308.2	1.044-1.038	[23]
	6	293.2-363.2	1.032-1.004	[37]
	4	288.2-318.2	1.048-1.031	[38]
	8	303-2-373.2	1.035-0.997	[39]
	10	298.2-343.2	1.040-1.014	[24]
	11	303.2-353.2	1.036-1.008	[40]
	7	303.2-333.2	1.037-1.020	[25]
**[C$_8$mim]Cl**	15	293.2-363.1	1.012-0.973	[20]
	4	298.2-363.2	1.01-0.976	[31]
	3	298.2-318.2	1.010-0.998	[34]
	7	293.2-353.2	1.012-0.977	[19]
	4	288.2-318.2	1.022-1.004	[38]
	10	298.2-343.2	1.009-0.983	[24]
	7	303.2-333.2	1.007-0.990	[25]
	10	283.2-363.2	1.019-0.974	[18]
	8	298.2-333.2	1.010-0.989	[21]
	10	278.2-343.2	1.021-0.983	[17]
**[C$_{10}$mim]Cl**	10	317.9-363.4	0.974-0.948	[28]
	8	298.2-363.2	0.983-0.943	[18]
	8	298.2-363.2	0.984-0.944	[41]

Table S6. Experimental and estimated densities, ρ, of pure IL as a function of temperature, at 0.1 MPa.

T / K	ρ / g·cm^{-3}			
	[C_8mim]Cl[a]	[C_8mim]Cl[b]	[C_{12}mim]Cl[b]	[C_4mim]Cl /[C_{12}mim]Cl equimolar mixture[b]
278.2	1.0212		0.9677	1.0075
283.2	1.0186 ± 0.0003			
288.2	1.0154			
293.2	1.0124 ± 0.0006	1.0152		
298.2	1.0101 ± 0.0013	1.0146	0.9677	1.0075
303.2	1.0069 ± 0.0007	1.0084	0.9635	1.0035
308.2	1.0037 ± 0.0005	1.0064		
313.2	1.0010 ± 0.0007	1.0026	0.9589	0.9993
318.2	0.9980 ± 0.0004	0.9999		
323.2	0.9953 ± 0.0008	0.9967	0.9528	0.9939
328.2	0.9919 ± 0.0002	0.9935		
333.2	0.9894 ± 0.0006	0.9907	0.9468	0.9881
338.2	0.9862 ± 0.0001	0.9889		
343.2	0.9833 ± 0.0001	0.9851	0.9407	0.9823
348.2	0.9812 ± 0.0001	0.9836		
353.2	0.9783 ± 0.0007	0.9799	0.9353	0.9768
358.2	0.9756	0.9771		
363.2	0.9733 ± 0.0003	0.9747	0.9291	0.9707

[a]Average values calculated from the experimental data included in **Table S5**. Standard deviation is placed after the plus-minus sign.

[b]Estimated values using the methodology proposed by Rebelo et al.[26]

Limiting partial molar excess properties

Table S7. Thermodynamic functions at infinite dilution, namely the partial molar excess Gibbs free energy ($\bar{G}_m^E$/kJ·mol^{-1}), partial molar excess enthalpy ($\bar{H}_m^E$/kJ·mol^{-1}), and partial molar excess entropy ($T_{ref}\bar{S}_m^E$/kJ·mol^{-1}) of water and organic solutes in [C_4mim]Cl,[16] [C_8mim]Cl, [C_{12}mim]Cl, and the equimolar [C4mim]Cl/ [C_{12}mim]Cl mixture.

**Solutes**	**[C_4mim]Cl[a]**			**[C_8mim]Cl**			**Equimolar mixture**			**[C_{12}mim]Cl**		
	$\bar{G}_m^E$	$\bar{H}_m^E$	$T_{ref}\bar{S}_m^E$	$\bar{G}_m^E$	$\bar{H}_m^E$	$T_{ref}\bar{S}_m^E$	$\bar{G}_m^E$	$\bar{H}_m^E$	$T_{ref}\bar{S}_m^E$	$\bar{G}_m^E$	$\bar{H}_m^E$	$T_{ref}\bar{S}_m^E$
	**T_{Ref} = 373.15 K**											
heptane	-	-	-	12.20	8.88	-3.32	9.95	5.08	-4.86	5.61	-1.57	-7.18
octane	17.57	9.94	-7.62	11.79	6.49	-5.30	9.73	2.93	-6.80	5.70	-1.14	-6.84
nonane	18.71	9.34	-9.38	11.58	3.69	-7.88	9.72	1.45	-8.27	6.02	-2.00	-8.02
decane	19.93	9.07	-10.86	11.72	3.04	-8.68	9.88	1.30	-8.58	6.27	-0.90	-7.17
cyclohexane	12.09	7.67	-4.42	9.80	14.55	4.75	7.86	11.74	3.87	4.03	2.18	-1.85
methylcyclohexane	13.44	7.42	-6.02	10.04	12.35	2.31	8.09	8.74	0.65	4.32	0.54	-3.78
benzene	4.78	-2.62	-7.40	2.26	0.01	-2.26	2.06	0.69	-1.37	0.82	-1.76	-2.58
toluene	6.47	-2.74	-9.21	3.25	-1.41	-4.66	3.03	-0.33	-3.37	1.60	-3.20	-4.80
ethylbenzene	8.06	-1.72	-9.77	4.41	-1.08	-5.49	3.90	-0.11	-4.00	2.26	-2.84	-5.09
p-xylene	8.17	-1.86	-10.03	4.27	-0.75	-5.02	3.99	-0.42	-4.41	2.34	-3.05	-5.39
methyl acetate	4.96	-2.17	-7.13	3.49	4.34	0.85	3.30	3.56	0.26	2.60	1.98	-0.63
ethyl acetate	6.85	-0.58	-7.43	4.52	3.30	-1.22	4.29	3.65	-0.64	3.17	1.14	-2.03
vinyl acetate	5.47	-2.54	-8.01	3.53	2.24	-1.29	3.44	2.33	-1.11	2.65	1.14	-1.51
THF	5.39	-0.24	-5.63	3.06	3.68	0.62	2.75	3.57	0.83	1.48	1.27	-0.22
1,4-dioxane	3.98	-2.92	-6.89	2.16	1.15	-1.01	2.10	1.57	-0.53	1.53	0.38	-1.15
diethyl ether	9.09	1.99	-7.10	8.70	9.94	1.24	7.22	9.22	2.00	4.38	3.72	-0.66
acetonitrile	0.55	-5.10	-5.65	-0.14	0.21	0.35	-0.08	0.11	0.19	0.35	1.34	0.99

pyridine	1.48	-7.27	-8.74	0.03	-0.94	-0.97	0.31	0.41	0.10	0.13	-0.12	-0.25
thiophene	2.47	-6.35	-8.82	0.53	-2.60	-3.13	0.46	-1.77	-2.23	-0.27	-3.15	-2.87
acetone	3.56	-2.59	-6.16	2.36	4.39	2.03	2.26	4.15	1.89	1.92	2.70	0.78
2-butanone	-	-	-	2.87	1.91	-0.96	2.79	2.53	-0.25	2.20	0.99	-1.21
methanol	-6.37	-9.42	-3.05	-7.62	-7.33	0.30	-7.52	-5.84	1.68	-7.51	-4.92	2.59
ethanol	-4.29	-8.17	-3.88	-6.22	-6.10	0.12	-6.09	-5.43	0.66	-6.36	-4.14	2.21
1-propanol	-3.40	-9.66	-6.26	-5.89	-7.90	-2.01	-5.78	-7.00	-1.21	-6.32	-6.10	0.22
2-propanol	-2.54	-9.87	-7.34	-5.01	-7.92	-2.91	-4.93	-6.90	-1.97	-5.47	-6.50	-1.02
isobutanol	-2.54	-9.77	-7.23	-5.64	-8.32	-2.67	-5.54	-7.24	-1.71	-6.31	-7.43	-1.13
1-butanol	-2.41	-9.35	-6.94	-5.50	-7.85	-2.36	-5.40	-7.14	-1.74	-6.20	-7.38	-1.17
2-butanol	-1.55	-10.28	-8.73	-4.66	-9.87	-5.21	-4.61	-8.90	-4.29	-5.33	-8.04	-2.71
tert-butanol	-0.80	-11.85	-11.05	-3.58	-10.98	-7.40	-3.84	-9.96	-6.13	-4.53	-8.10	-3.57
water	-9.14	-13.93	-4.79	-8.98	-11.28	-2.30	-8.88	-11.08	-2.20	-8.98	-11.79	-2.81

[a]The partial molar thermodynamic functions of water and organic solutes in [C_4mim]Cl were calculated from the γ_{13}^{∞} (interpolated at 373.15 K) reported by Martins et al.[16]

For most organic solutes, an increase in the IL alkyl chain leads to lower Gibbs free energy values, except for acetonitrile where the minimum $\bar{G}_m^E$ is found in [C_8mim]Cl. Regarding water, the lowest $\bar{G}_m^E$ is observed in [C_4mim]Cl, and no significant differences are observed in the other pure IL. In general, negative $\bar{G}_m^E$ were registered for alcohols, water, acetonitrile (in [C_8mim]Cl and in the mixture), and thiophene (only in [C_{12}mim]Cl), whereas positive values were found for the other solutes.

Regarding the entropic contribution, $T_{ref}\bar{S}_m^E$, the values increase as the IL cation length increases for alcohols and decane. For the other compounds, in general, the $T_{ref}\bar{S}_m^E$ product ranks as: [C_8mim]Cl ~ equimolar mixture > [C_{12}mim]Cl > [C_4mim]Cl. For most of the studied solutes, negative values were found for $T_{ref}\bar{S}_m^E$ product.

In the case of the partial molar enthalpies, the values increase as the IL cation alkyl chain length decreases for alkanes, whereas the opposite behavior is observed for alcohols and nitrogen compounds. For cycloalkanes and aromatic hydrocarbons (except benzene), the highest $\bar{H}_m^E$ values were observed in [C_8mim]Cl or in the equimolar mixture, followed by [C_4mim]Cl and [C_{12}mim]Cl, respectively. For the remaining solutes, the highest $\bar{H}_m^E$ are also observed in [C_8mim]Cl or in the equimolar mixture, but [C_{12}mim]Cl delivers higher enthalpic contributions than [C_4mim]Cl.

The differences between the partial molar energies, in pure [C_8mim]Cl and in the equimolar mixture are more pronounced in the low polar solutes (e.g., alkanes and cycloalkanes) than in more polar solutes (e.g., acetonitrile, alcohols, and water).

Since $\bar{H}_m^E$ can be directly obtained from the slope of $\ln(\gamma_{13}^{\infty})$ vs $1/T$ (**Eq. S5**), this property solely gives some insights about the solute-solvent affinity. For example, the positive sign of $\bar{H}_m^E$ for most alkanes and diethyl ether in the four solvents indicate that solute-IL interactions increase as the temperature increases. The opposite behavior is observed for alcohols, water, thiophene and most of the aromatics, where negative $\bar{H}_m^E$ values were registered. For cyclic ethers, esters and ketones, the affinity between the solute and [C_4mim]Cl decreases with temperature (negative $\bar{H}_m^E$ values), whereas positive enthalpic contributions are observed for these solutes in the other studied ionic liquids.

To further explore this topic, the partial molar thermodynamic functions were represented as function of $\ln(\gamma_{13}^{\infty})$ where four distinct regions can be distinguished – **Figure S3**.

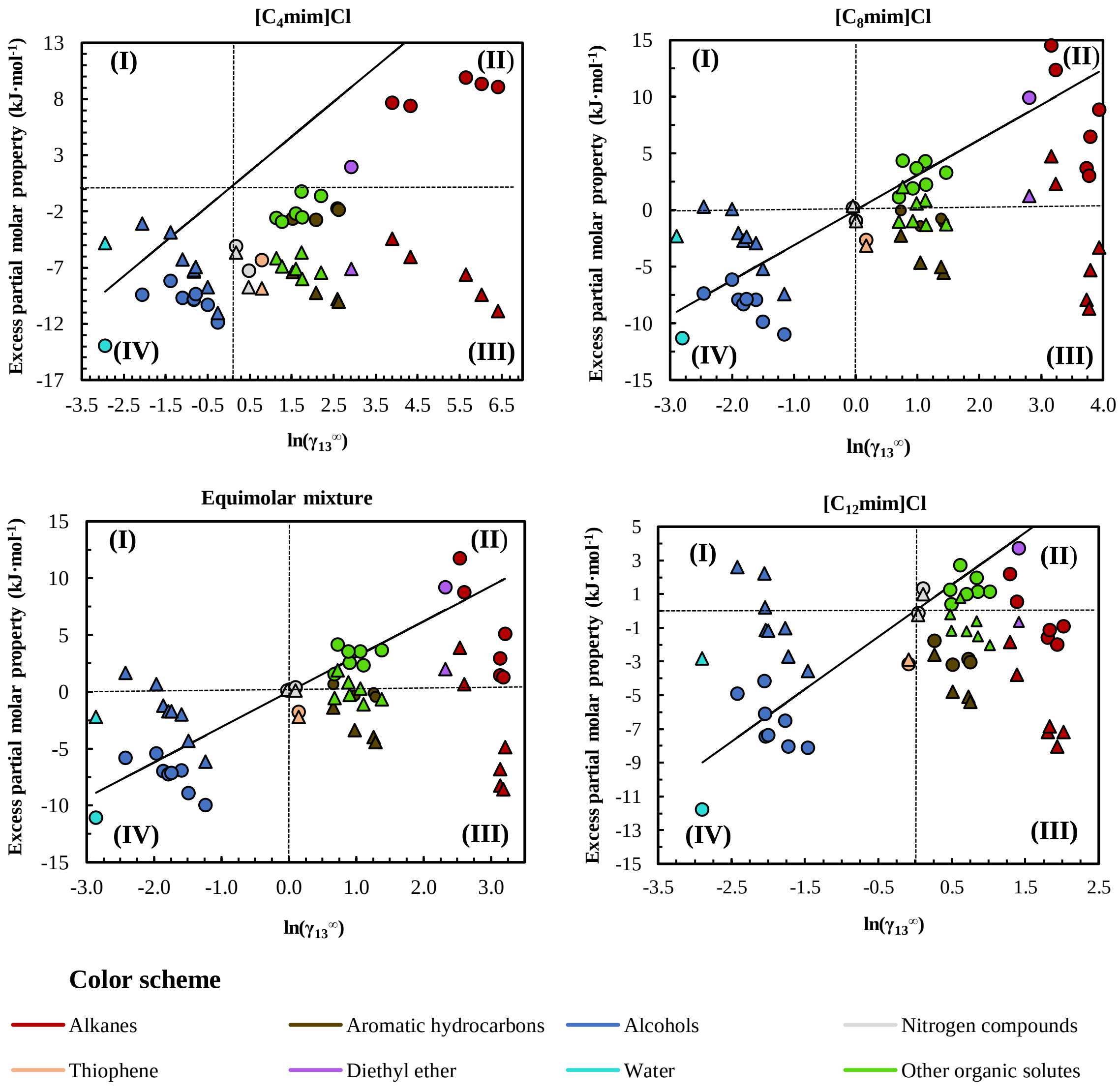

Figure S3. Partial molar excess properties as a function of $\ln(\gamma_{13}^{\infty})$ for the studied imidazolium chloride IL at 373.15 K. The line represents $\bar{G}_m^E$, the circles correspond to $\bar{H}_m^E$, and the triangles stand for $T_{ref}\bar{S}_m^E$.

As can be seen from **Figure S3**, particularly for alcohols and water, the partial molar excess properties and the activity coefficients at infinite dilution are generally negative, resulting in a distribution of the thermodynamic energies throughout region IV. A few exceptions are observed for the $T_{ref}\bar{S}_m^E$ values of some primary alcohols in [C8mim]Cl, [C12mim]Cl, and in the equimolar mixture, which fall in region I. The low γ_{13}^{∞} values lead to negative $\bar{G}_m^E$ for all these solutes, suggesting that their solvation in the imidazolium-chloride IL is thermodynamic favorable. Besides, the observed $\bar{H}_m^E$ values are lower than the correspondent $T_{ref}\bar{S}_m^E$ values, revealing there is a dominant enthalpic effect on the solvation of alcohols and water, which are polar protic solutes.

On the other hand, the positive $\ln(\gamma_{13}^{\infty})$ values observed (and consequently $\bar{G}_m^E$) for alkanes and diethyl ether, combined with the often negative $T_{ref}\bar{S}_m^E$ values indicate that the solvation of these solutes in the studied IL is highly unfavorable. In the case of [C_4mim]Cl, [C_8mim]Cl, and the equimolar [C_4mim]Cl/[C_{12}mim]Cl mixture, the high $\bar{H}_m^E$ values show an enthalpic dominant effect in the solvation of diethyl ether, cycloalkanes, and heptane, while the entropic effects are more relevant in the solvation of the larger linear alkanes. In fact, the IL alkyl chain size seems to play a crucial role in the solvation of low polar aliphatic hydrocarbons in the abovementioned IL, where an increase in the alkyl chain leads to a decrease in the enthalpic and entropic contributions. Nevertheless, this trend is not systematically observed in [C_{12}mim]Cl, where the entropic effects generally dominate over the enthalpic ones in the solvation of alkanes. Differently from the other studied IL, the partial molar excess enthalpies of the linear alkanes are negative in [C_{12}mim]Cl (falling in region III), suggesting that larger cation alkyl chains might favor solvation of these solutes by significantly reducing the $\bar{H}_m^E$, while delivering similar $T_{ref}\bar{S}_m^E$ values.

Regarding the aromatic hydrocarbons, positive deviations from the ideality are observed along with generally negative $\bar{H}_m^E$ and $T_{ref}\bar{S}_m^E$ values, resulting in their main distribution within region III, where the entropic effects are dominant. For this family of solutes, the lowest $T_{ref}\bar{S}_m^E$ values are registered in [C_4mim]Cl,[16] while the highest values are observed in the equimolar mixture. In the case of thiophene and the nitrogen compounds, acetonitrile and pyridine, the partial excess Gibbs energies are close to zero, because of the similar enthalpic and entropic contributions.

Lastly, the partial molar excess energies of the other organic solutes, comprising ketone, esters, and cyclic ethers, are mainly distributed in regions II and III. In [C_4mim]Cl, the strong entropy losses prevail over the enthalpic ones, resulting in positive $\bar{G}_m^E$ values. On the other hand, the endothermic interactions are predominant in the solvation of these aprotic polar solutes in [C_8mim]Cl and the equimolar mixture, whereas the enthalpic and entropic contributions cancel each other in [C_{12}mim]Cl. Besides, it is worth mentioning that most of the partial molar excess enthalpies (79%) and entropies (100%) are negative in [C_4mim]Cl, while significantly lower $\bar{G}_m^E$ values were achieved in [C_4mim]Cl compared to the other IL, particularly for low polar solutes, such as alkanes and cycloalkanes.

Selectivities and Capacities

Table S8. Overview of the available experimental S_{ij}^{∞}, k_j^{∞}, and Q_{ij}^{∞} of octane/benzene and cyclohexane/benzene mixtures in chloride-based IL and important industrial solvents, at 333.15 K.

**Solvent**	$\boldsymbol{S_{ij}^{\infty}/\ k_j^{\infty}/Q_{ij}^{\infty}}$		**Reference**
	**octane/benzene**	**cyclohexane/benzene**	
[C_4mim]Cl	100.37/0.24/24.09	15.72/0.24/3.77[a]	16
[C_6mim]Cl	32.83/0.33/10.83	9.44/0.33/3.12	37
[C_8mim]Cl	27.91/0.48/13.40	19.80 /0.48/9.50	this work
[C_4mim]Cl/[C_{12}mim]Cl equimolar mixture	13.06/0.50/6.53	10.34 /0.50/5.17	this work
[C_{12}mim]Cl	4.91/0.82/4.03	3.27/0.82/2.68	this work
[$P_{6,6,6,14}$]Cl	3.55/2.02/7.17	2.20/2.02/4.44	42
Sulfolane	34.84/0.43/14.98	-	43
	-	2.13/0.34/0.72	44
n-methyl pyrrolidone (NMP)	12.51/0.91/11.38	5.75/0.91/5.23	45
	-	7.86/1.01/7.94	46
n-formyl morpholine (NFM)	20.65/0.52/10.74	7.21/0.52/3.75	45
	-	9.08/0.49/4.45	46

[a]Extrapolated using the data reported by the authors.

Table S9. Overview of the available experimental S_{ij}^{∞}, k_j^{∞}, and Q_{ij}^{∞} of octane/pyridine and octane/thiophene mixtures in chloride-based ionic liquids, at 333.15 K.

Ionic liquid	S_{ij}^{∞}/ k_j^{∞} / Q_{ij}^{∞}		Reference
	octane/pyridine	octane/thiophene	
$[C_4mim]Cl$	347.97/0.82/285.34	243.46/0.58/141.21[a]	[16]
$[C_8mim]Cl$	59.48/1.03/61.26	53.63/0.93/49.88	this work
$[C_4mim]Cl/[C_{12}mim]Cl$ equimolar mixture	23.17/0.89/20.62	23.96/0.92/22.04	this work
$[C_{12}mim]Cl$	5.77/0.97/5.60	7.38/1.24/9.15	this work
$[P_{6,6,6,14}]Cl$	4.17/2.38/9.92	4.93/2.81/13.85	[42]

[a]Extrapolated using the data reported by the authors.

Table S10. Overview of the available experimental S_{ij}^{∞}, k_j^{∞}, and Q_{ij}^{∞} of ethanol/water, 2-propanol/water, and acetone/methanol azeotropic mixtures in chloride-based ionic liquids, at 333.15 K.[a]

Ionic liquid	$S_{ij}^{\infty}/ k_j^{\infty} /Q_{ij}^{\infty}$			Reference
	**ethanol/water**	**2-propanol/water**	**acetone/methanol**	
[C_4mim]Cl	5.96/32.58/194.18	9.82/32.58/319.97	32.02/11.22/359.26	[16]
[C_6mim]Cl	-	-	17.98/7.37/132.51	[37]
[C_8mim]Cl	2.98/27.99/83.41	4.07/27.99/113.98	33.36/15.60/520.42	this work
[C_4mim]Cl/[C_{12}mim]Cl equimolar mixture	3.05/27.07/82.56	4.19/27.07/113.47	34.70/14.20/492.74	this work
[C_{12}mim]Cl	2.91/26.58/77.35	3.47/26.58/92.29	28.45/13.76/391.47	this work
[$P_{6,6,6,14}$]Cl	6.64/32.13/213.34[b]	5.70/27.59/157.34[b]	32.93/41.42/1363.96	[42]

[a]Extrapolated using the data reported by the authors.
[b]The activity coefficient at infinite dilution of water is higher than the values of the alcohol in this IL. Therefore, the k_j^{∞} value stands for the alcohol.

COSMO-RS

Table S11. Summary of the selectivities and capacities, at 333.15 K, in imidazolium-chloride based IL with different cations obtained with the COSMO-RS model using both TZVP and TZVPD-FINE quantum chemical levels.

Ionic liquid	S_{ij}^{∞} / k_j^{∞}													
	**octane/benzene**		**cyclohexane/benzene**		**octane/pyridine**		**octane/thiophene**		**ethanol/water**		**2-propanol/water**		**acetone/methanol**	
	TZVP	TZVPD-FINE	TZVP	TZVPD-FINE	TZVP	TZVPD-FINE	TZVP	TZVPD-FINE	TZVP	TZVPD-FINE	TZVP	TZVPD-FINE	TZVP	TZVPD-FINE
[C_2mim]Cl	3.2/2.8	104.0/0.2	2.5/2.8	10.7/0.2	3.9/3.4	195.0/0.5	5.9/5.1	252.1/0.6	1.1/12.2	1.5/1.2	1.2/12.2	2.6/1.2	5.8/10.7	2.4/1.6
[C_4mim]Cl	7.4/1.1	52.5/0.4	3.1/1.1	7.7/0.4	10.7/1.5	92.8/0.6	13.3/1.9	108.4/0.7	1.6/10.8	1.1/1.0	2.2/10.8	1.7/1.0	6.5/7.6	1.7/1.6
[C_6mim]Cl	9.6/0.8	29.8/0.5	3.3/0.8	5.9/0.5	14.6/1.2	50.4/0.8	16.2/1.3	54.6/0.9	2.5/9.8	1.2/0.9	2.5/9.8	1.2/0.8	6.2/6.7	1.2/1.5
[C_8mim]Cl	9.5/0.7	18.9/0.6	3.2/0.7	4.8/0.6	14.6/1.1	31.2/1.0	15.2/1.2	31.5/1.0	2.4/9.0	1.5/0.9	2.4/9.0	1.2/0.7	5.7/6.4	1.0/1.4
[C_{12}mim]Cl	7.4/0.8	9.7/0.9	2.7/0.8	3.5/0.9	10.9/1.2	15.3/1.4	10.7/1.2	14.2/1.3	1.4/7.8	2.2/0.9	2.0/7.8	2.0/0.8	4.8/6.3	1.5/1.9

Table S12. Overview of the selectivities and capacities at 333.15 K and in [C_4mim]Cl/[C_{12}mim]Cl mixtures at different molar proportions, obtained with the COSMO-RS model using both TZVP and TZVPD-FINE quantum chemical levels.

[C_4mim]Cl/[C_{12}mim]Cl molar proportion	S_{ij}^{∞} / k_j^{∞}													
	**octane/benzene**		**cyclohexane/benzene**		**octane/pyridine**		**octane/thiophene**		**ethanol/water**		**2-propanol/water**		**acetone/methanol**	
	TZVP	TZVPD-FINE	TZVP	TZVPD-FINE	TZVP	TZVPD-FINE	TZVP	TZVPD-FINE	TZVP	TZVPD-FINE	TZVP	TZVPD-FINE	TZVP	TZVPD-FINE
1:0	7.4/1.1	52.5/0.4	3.1/1.1	7.7/0.4	10.7/1.5	92.8/0.6	13.3/1.9	108.4/0.7	1.6/10.8	1.1/1.0	2.2/10.8	1.7/1.0	6.5/7.6	1.7/1.6
3:1	9.6/0.8	27.0/0.5	3.3/0.8	5.7/0.5	14.7/1.2	45.7/0.8	16.3/0.9	48.3/0.9	1.7/9.7	1.2/1.0	2.5/9.7	1.1/0.7	6.2/6.7	1.2/1.4
1:1	9.5/0.7	17.3/0.6	3.2/0.7	4.6/0.6	14.6/1.1	28.7/1.0	15.2/1.2	28.2/1.0	1.7/9.0	1.5/0.9	2.4/9.0	1.2/0.7	5.7/6.4	1.1/1.5
1:3	8.5/0.8	12.5/0.7	2.9/0.7	4.0/0.7	12.8/1.1	20.2/1.2	12.9/1.1	31.5/1.0	1.5/8.3	1.9/0.9	2.2/8.3	1.6/0.8	6.2/6.3	1.3/1.7
0:1	7.4/0.8	9.7/0.9	2.7/0.8	3.5/0.9	10.9/1.2	15.3/1.4	10.7/1.2	14.2/1.3	1.4/7.8	2.2/0.9	2.0/7.8	2.0/0.8	4.8/6.3	1.5/1.9

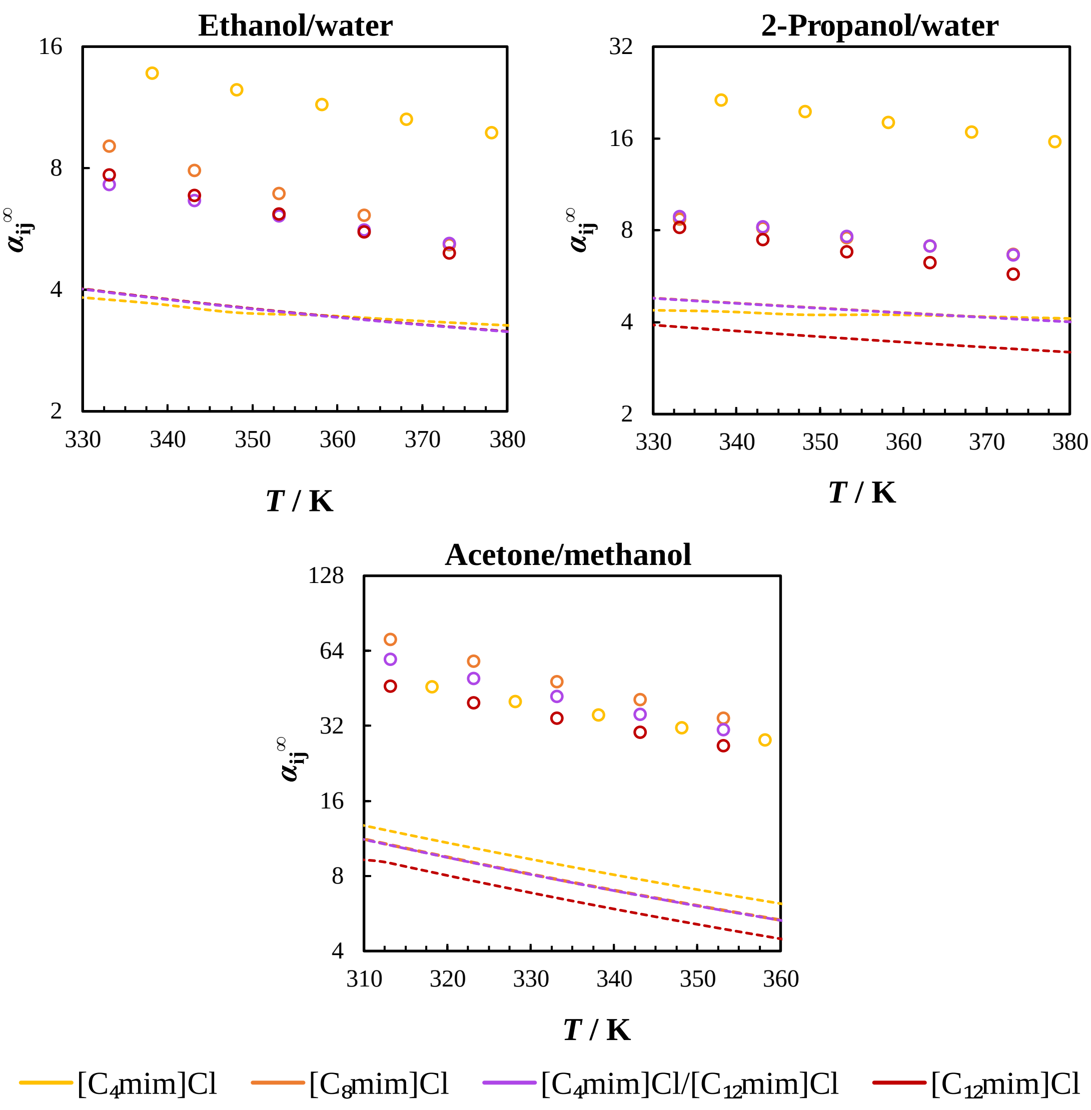

Figure S4. Comparison of experimental and predicted α_{ij}^{∞} (at 101.3 kPa) for ethanol/water, 2-propanol/water and acetone/methanol mixtures in the presence of the imidazolium chloride IL. The open circles (○) represent the experimental data calculated from the results obtained in this work and reported by Martins et al,[16] and the dotted lines depict the COSMO-RS predictions using the TZVP parameterization set. α_{ij}^{∞} was calculated using the pure compounds' vapor pressures collected from the DIPPR database.[5]

Table S13. Overview of the experimental and predicted (with COSMO-RS) relative volatilities at infinite dilution, α_{ij}^{∞}, of ethanol/water, 2-propanol/water, and acetone/methanol azeotropes in the presence of different entrainers. α_{ij}^{∞} was calculated using the pure compounds' vapor pressures collected from the DIPPR database.[5]

**Azeotropic mixture**	**Entrainer**	***T* / K**	**Experimental α_{ij}^{∞}**	**Predicted α_{ij}^{∞}**
**Ethanol/water**	[C_4mim]Cl	338.2	13.77[a]	3.70
		348.2	12.56[a]	3.51
		358.2	11.52[a]	3.46
		368.2	10.62[a]	3.36
		378.2	9.83[a]	3.28
	[C_8mim]Cl	333.2	9.08	3.94
		343.2	7.91	3.72
		353.2	6.94	3.54
		363.2	6.14	3.38
		373.2	5.18	3.24
	[C_4mim]Cl/[C_{12}mim]Cl equimolar mixture	333.2	7.30	3.94
		343.2	6.67	3.72
		353.2	6.12	3.53
		363.2	5.64	3.37
		373.2	5.23	3.23
	[C_{12}mim]Cl	333.2	7.71	3.94
		343.2	6.88	3.72
		353.2	6.18	3.54
		363.2	5.58	3.38
		373.2	4.95	3.24
**2-Propanol/water**	[C_4mim]Cl	338.2	21.44[a]	4.34
		348.2	19.69[a]	4.23
		358.2	18.16[a]	4.23
		368.2	16.83[a]	4.18
		378.2	15.66[a]	4.13
	[C_8mim]Cl	333.2	8.76	4.75
		343.2	8.15	4.57
		353.2	7.61	4.41
		363.2	7.14	4.25
		373.2	6.69	4.11
	[C_4mim]Cl/[C_{12}mim]Cl equimolar mixture	333.2	8.93	4.73
		343.2	8.25	4.56
		353.2	7.66	4.39
		363.2	7.14	4.24
		373.2	6.67	4.10
	[C_{12}mim]Cl	333.2	8.19	3.86
		343.2	7.47	3.69
		353.2	6.85	3.54
		363.2	6.31	3.40
		373.2	5.76	3.27

	[C4mim]Cl	318.2	45.95[a]	11.19
		328.2	40.20[a]	9.60
		338.2	35.44[a]	8.30
		348.2	31.48[a]	6.76
		358.2	28.12[a]	6.34
	[C8mim]Cl	313.2	71.11	10.66
		323.2	58.32	9.05
		333.2	48.25	7.76
		343.2	40.77	6.70
**Acetone/methanol**		353.2	34.42	5.83
	[C4mim]Cl/[C12mim]Cl equimolar mixture	313.2	59.30	10.63
		323.2	49.64	9.03
		333.2	42.01	7.73
		343.2	35.67	6.68
		353.2	30.94	5.81
	[C12mim]Cl	313.2	46.19	9.01
		323.2	39.76	7.64
		333.2	34.46	6.53
		343.2	30.16	5.64
		353.2	26.78	4.90

[a]Calculated using the activity coefficients reported by Martins et al.[16]

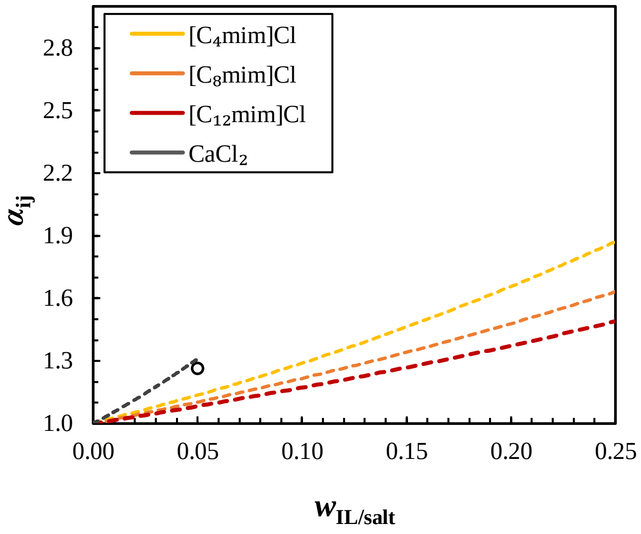

Figure S5. Overview of available experimental and predicted α_{ij} (at 101.3 kPa) for acetone/methanol mixture in the presence of imidazolium chloride IL and calcium chloride. Open circles represent the experimental datum available for $CaCl_2$[47] and dotted lines depict the COSMO-RS predictions using the TZVP parameterization. $CaCl_2$ predictions were removed for $w_{IL/Salt} > 0.05$ due to experimental evidence of saturation of the liquid phase.[47]

Table S14. Overview of the experimental and predicted (COSMO-RS) relative volatilities (at 101.3 kPa) of ethanol/water, 2-propanol/water, and acetone/methanol azeotropes in the presence of different entrainers. COSMO-RS predictions were performed with the TZVP parameterization.

Azeotropic mixture	Entrainer	$w_{IL/Salt}$	α_{ij}[a,b]	Source
**Ethanol/water**	$CaCl_2$	0.05	1.57	Experimental[48]
		0.10	2.12	
		0.15	2.92	
		0.05	1.14	Predicted with COSMO-RS
		0.10	1.30	
		0.15	1.50	
		0.20	1.74	
		0.25	2.04	
	$[C_4mim]Cl$	0.1	1.09	Experimental[49]
		0.2	1.25	
		0.20	1.34	Experimental[50]
		0.10	1.20	Experimental[51,c]
		0.15	1.35	
		0.05	1.05	Predicted with COSMO-RS
		0.10	1.10	
		0.15	1.16	
		0.20	1.23	
		0.25	1.30	
	$[C_8mim]Cl$[d]	0.05	1.03	Predicted with COSMO-RS
		0.10	1.07	
		0.15	1.11	
		0.20	1.15	
		0.25	1.20	
	$[C_{12}mim]Cl$	0.05	1.02	Predicted with COSMO-RS
		0.10	1.05	
		0.15	1.08	
		0.20	1.11	
		0.25	1.14	
**2-Propanol/water**	$CaCl_2$	0.05	1.57	Experimental[52]
		0.10	1.67	
		0.15	1.61	
		0.01	1.23	Experimental[53]
		0.02	1.22	
		0.02	1.22	
		0.03	1.34	
		0.05	1.49	
		0.06	1.49	
		0.01	1.04	Predicted with COSMO-RS
		0.02	1.09	
		0.02	1.13	
		0.03	1.17	
		0.05	1.22	
		0.06	1.26	
		0.10	1.45	
		0.15	1.77	
		0.20	2.18	
		0.25	2.73	
**2-Propanol/water**	$[C_4mim]Cl$	0.10	1.35	Experimental[54]

**2-Propanol/water**	[C_4mim]Cl	0.05	1.08	Predicted with COSMO-RS
		0.10	1.16	
		0.15	1.25	
		0.20	1.36	
		0.25	1.48	
	[C_8mim]Cl[d]	0.05	1.05	Predicted with COSMO-RS
		0.10	1.11	
		0.15	1.17	
		0.20	1.24	
		0.25	1.31	
	[C_{12}mim]Cl	0.05	1.04	Predicted with COSMO-RS
		0.10	1.08	
		0.15	1.12	
		0.20	1.16	
		0.25	1.22	
**Acetone/methanol**	$CaCl_2$	0.05	1.27	Experimental[47]
		0.05	1.31	Predicted with COSMO-RS[e]
	[C_4mim]Cl	0.05	1.14	Predicted with COSMO-RS
		0.10	1.29	
		0.15	1.47	
		0.20	1.66	
		0.25	1.87	
	[C_8mim]Cl[d]	0.05	1.10	Predicted with COSMO-RS
		0.10	1.22	
		0.15	1.34	
		0.20	1.48	
		0.25	1.63	
	[C_{12}mim]Cl	0.05	1.08	Predicted with COSMO-RS
		0.10	1.17	
		0.15	1.27	
		0.20	1.37	
		0.25	1.49	

[a]The predicted α_{ij}values were calculated using Eq. (S12), $\alpha_{ij} = \frac{\gamma_i p_i^0}{\gamma_j p_j^0}$, where the activity coefficients were estimated using COSMO-RS and the vapor pressures were collected from the DIPPR 801 database.[5]
[b]Predicted α_{ij}calculated using as reference: $\alpha_{ij} = 1$ when $w_{IL/Salt} = 0$.
[c]Interpolated at the azeotropic composition (free of IL) from the available experimental data.
[d]Predicted α_{ij} values for [C_4mim]Cl/[C_{12}mim]Cl equimolar mixture are the same as those predicted for [C_8mim]Cl.
[e]Predictions were truncated at $w_{IL/Salt} = 0.05$ due to evidence of the saturation point in the liquid phase.[47]

References

(1) Domańska, U.; Bogel-Łukasik, E.; Bogel-Łukasik, R. 1-Octanol/Water Partition Coefficients of 1-Alkyl-3-Methylimidazolium Chloride. *Chem. - A Eur. J.* **2003**, *9* (13), 3033–3041.

(2) Yaws, C. L. *Thermophysical Properties of Chemicals and Hydrocarbons*, 2nd ed.; Elsevier Inc., 2014.

(3) Everett, D. H. Effect of Gas Imperfection on G.L.C. Measurements: A Refined Method for Determining Activity Coefficients and Second Virial Coefficients. *Trans. Faraday Soc.* **1965**, *61* (4), 1637–1645.

(4) Cruickshank, A. J. B.; Gainey, B. W.; Hicks, C. P.; Letcher, T. M.; Moody, R. W.; Young, C. L. Gas-Liquid Chromatographic Determination of Cross-Term Second Virial Coefficients Using Glycerol. Benzene + Nitrogen and Benzene + Carbon Dioxide at 50°C. *Trans. Faraday Soc.* **1969**, *65*, 1014–1031.

(5) (U.S.), D. I. for P. P. D.; Engineers., A. I. of C.; (U.S.), N. I. of S. and T. DIPPR Project 801 – Full Version. Design Institute for Physical Property Research/AIChE. BYU DIPPR, Thermophysical Properties Laboratory: Provo, UT 2010.

(6) Tsonopoulos, C. Second Virial Cross-Coefficients: Correlation and Prediction of k Ij. In *Equations of State in Engineering and Research*; Chao, K. C., Jr, R. L. R., Eds.; American Chemical Society, 1979; pp 143–162.

(7) Poling, B. E.; Prausnitz, J. M.; O'Connel, J. P. *The Properties of Gases and Liquids*, 5th ed.; McGraw-Hill, 2001; Vol. 1.

(8) Blumberg, L. M. Properties of James-Martin Compressibility Correction Factor. *Chromatographia* **1997**, *44* (5–6), 326–329.

(9) Marciniak, A. Influence of Cation and Anion Structure of the Ionic Liquid on Extraction Processes Based on Activity Coefficients at Infinite Dilution. A Review. *Fluid Phase Equilib.* **2010**, *294* (1–2), 213–233.

(10) Kossack, S.; Kraemer, K.; Gani, R.; Marquardt, W. A Systematic Synthesis Framework for Extractive Distillation Processes. *Chem. Eng. Res. Des.* **2008**, *86* (7), 781–792.

(11) Órfão, E. F.; Dohnal, V.; Blahut, A. Infinite Dilution Activity Coefficients of Volatile Organic Compounds in Two Ionic Liquids Composed of the Tris(Pentafluoroethyl)Trifluorophosphate ([FAP]) Anion and a Functionalized Cation. *J. Chem. Thermodyn.* **2013**, *65*, 53–64.

(12) Boussaha, M.; Khimeche, K.; Dahmani, A. Activity Coefficients at Infinite Dilution for Hydrocarbons in Fatty Alcohols Determined by Gas-Liquid Chromatography. *J. Chem. Eng. Data* **2010**, *56*, 850–858.

(13) Lei, Z.; Chen, B.; Ding, Z. Thermodynamic Fundamentals. In *Special Distillation Processes*; Elsevier, 2005; pp 1–58.

(14) Pereiro, A. B.; Araújo, J. M. M.; Esperança, J. M. S. S.; Marrucho, I. M.; Rebelo, L. P. N. Ionic Liquids in Separations of Azeotropic Systems - A Review. *J. Chem. Thermodyn.* **2012**, *46*, 2–28.

(15) Brouwer, T.; Kersten, S. R. A.; Bargeman, G.; Schuur, B. Solvent Pre-Selection for Extractive Distillation Using Infinite Dilution Activity Coefficients and the Three-Component Margules Equation. *Sep. Purif. Technol.* **2021**, *276*, 119230.

(16) Martins, M. A. R.; Coutinho, J. A. P.; Pinho, S. P.; Domańska, U. Measurements of Activity Coefficients at Infinite Dilution of Organic Solutes and Water on Polar Imidazolium-Based Ionic Liquids. *J. Chem. Thermodyn.* **2015**, *91*, 194–203.

(17) Singh, T.; Kumar, A. Temperature Dependence of Physical Properties of Imidazolium Based Ionic Liquids: Internal Pressure and Molar Refraction. *J. Solution Chem.* **2009**, *38* (8), 1043–1053.

(18) Yousefi, M.; Naseri, A.; Abdouss, M.; Miran Beigi, A. A. Synthesis and Characterization of Eight Hydrophilic Imidazolium-Based Ionic Liquids and Their Application on Enhanced Oil Recovery. *J. Mol. Liq.* **2017**, *248*, 370–377.

(19) Tomida, D.; Kenmochi, S.; Qiao, K.; Yokoyama, C. Densities and Thermal Conductivities of Ionic Liquids, 1-Hexyl-3-Methylimidazolium Chloride, 1-Octyl-3-Methylimidazolium Chloride, and 1-Octyl-3-Methylimidazolium Bromide, at Pressures up to 20 MPa. *High Temp. - High Press.* **2017**, *46* (2), 101–114.

(20) Dowell, N. Mac; Llovell, F.; Sun, N.; Hallet, J. P.; George, A.; Hunt, P. A.; Welton, T.; Simmons, B. A.; Vega, L. F. New Experimental Density Data and Soft-SAFT Models of Alkylimidazolium ($[C_nC_1im]^+$) Chloride (Cl^-), Methylsulfate ($[MeSO_4]^-$), and Dimethylphosphate ($[Me_2PO_4]^-$) Based Ionic Liquids. *J. Phys. Chem. B* **2014**, *118*, 6206–6221.

(21) Altuwaim, M. S.; Alkhaldi, K. H. A. E.; Al-Jimaz, A. S.; Mohammad, A. A. Temperature Dependence of Physicochemical Properties of Imidazolium-, Pyroldinium-, and Phosphonium-Based Ionic Liquids. *J. Chem. Eng. Data* **2014**, *59* (6), 1955–1963.

(22) Seddon, K. R.; Stark, A.; Torres, M. J. Viscosity and Density of 1-Alkyl-3-

Methylimidazolium Ionic Liquids. In *ACS Symposium Series*; 2002; Vol. 819, pp 34–49.

(23) Sastry, N. V.; Vaghela, N. M.; Macwan, P. M. Densities, Excess Molar and Partial Molar Volumes for Water + 1-Butyl- or, 1-Hexyl- or, 1-Octyl-3-Methylimidazolium Halide Room Temperature Ionic Liquids at T = (298.15 and 308.15) K. *J. Mol. Liq.* **2013**, *180*, 12–18.

(24) Gómez, E.; González, B.; Domínguez, Á.; Tojo, E.; Tojo, J. Dynamic Viscosities of a Series of 1-Alkyl-3-Methylimidazolium Chloride Ionic Liquids and Their Binary Mixtures with Water at Several Temperatures. *J. Chem. Eng. Data* **2006**, *51* (2), 696–701.

(25) Ning, H.; Hou, M. Q.; Mei, Q. Q.; Liu, Y. H.; Yang, D. Z.; Han, B. X. The Physicochemical Properties of Some Imidazolium-Based Ionic Liquids and Their Binary Mixtures. *Sci. China Chem.* **2012**, *55* (8), 1509–1518.

(26) Rebelo, L. P. N.; Najdanovic-Visak, V.; Azevedo, R. G. de; Esperança, J. M. S. S.; Ponte, M. N. da; Guedes, H. J. R.; Visak, Z. P.; Sousa, H. C. de; Szydlowski, J.; Lopes, J. N. C.; Cordeiro, T. C. *Ionic Liquids III A: Fundamentals, Progress, Challenges, and Ppportunities*; Rogers, R. D., Seddon, K. R., Eds.; ACS Symposium Series; American Chemical Society: Washington, DC, 2005; Vol. 901.

(27) Martins, M. A. R.; Vilas-Boas, S. M.; Cordova, I. W.; Carvalho, P. J.; Domańska, U.; Ferreira, O.; Coutinho, J. A. P.; Pinho, S. P. Infinite Dilution Activity Coefficients in the Smectic and Isotropic Phases of Tetrafluoroborate-Based Ionic Liquids. *J. Chem. Eng. Data* **2021**, *66* (6), 2587–2596.

(28) Součková, M.; Klomfar, J.; Pátek, J. Group Contribution and Parachor Analysis of Experimental Data on Density and Surface Tension for Members of the Homologous Series of 1-C_n-3-Methylimidazolium Chlorides. *Fluid Phase Equilib.* **2017**, *454*, 43–56.

(29) Hiraga, Y.; Koyama, K.; Sato, Y.; Smith, R. L. High Pressure Densities for Mixed Ionic Liquids Having Different Functionalities: 1-Butyl-3-Methylimidazolium Chloride and 1-Butyl-3-Methylimidazolium Bis(Trifluoromethylsulfonyl)Imide. *J. Chem. Thermodyn.* **2017**, *108*, 7–17.

(30) Machida, H.; Taguchi, R.; Sato, Y.; Smith, R. L. Measurement and Correlation of High Pressure Densities of Ionic Liquids, 1-Ethyl-3-Methylimidazolium L-Lactate ([Emim][Lactate]), 2-Hydroxyethyl- Trimethylammonium L-Lactate ([(C_2H_4OH)(CH_3)$_3$N][Lactate]), and 1-Butyl-3-Methylimidazolium Chloride

([Bmim]. *J. Chem. Eng. Data* **2011**, *56* (4), 923–928.

(31) He, R. H.; Long, B. W.; Lu, Y. Z.; Meng, H.; Li, C. X. Solubility of Hydrogen Chloride in Three 1-Alkyl-3-Methylimidazolium Chloride Ionic Liquids in the Pressure Range (0 to 100) KPa and Temperature Range (298.15 to 363.15) K. *J. Chem. Eng. Data* **2012**, *57* (11), 2936–2941.

(32) Govinda, V.; Attri, P.; Venkatesu, P.; Venkateswarlu, P. Thermophysical Properties of Dimethylsulfoxide with Ionic Liquids at Various Temperatures. *Fluid Phase Equilib.* **2011**, *304* (1–2), 35–43.

(33) Vieira, N. S. M.; Vázquez-Fernández, I.; Araújo, J. M. M.; Plechkova, N. V.; Seddon, K. R.; Rebelo, L. P. N.; Pereiro, A. B. Physicochemical Characterization of Ionic Liquid Binary Mixtures Containing 1-Butyl-3-Methylimidazolium as the Common Cation. *J. Chem. Eng. Data* **2019**, *64* (11), 4891–4903.

(34) Kumar, B.; Singh, T.; Rao, K. S.; Pal, A.; Kumar, A. Thermodynamic and Spectroscopic Studies on Binary Mixtures of Imidazolium Ionic Liquids in Ethylene Glycol. *J. Chem. Thermodyn.* **2012**, *44* (1), 121–127.

(35) Li, J.; Zhu, H.; Peng, C.; Liu, H. Densities and Viscosities for Ionic Liquids [BMIM][BF_4] and [BMIM][Cl] and Their Binary Mixtures at Various Temperatures and Atmospheric Pressure. *Chinese J. Chem. Eng.* **2019**, *27* (12), 2994–2999.

(36) Kavitha, T.; Vasantha, T.; Venkatesu, P.; Rama Devi, R. S.; Hofman, T. Thermophysical Properties for the Mixed Solvents of N-Methyl-2-Pyrrolidone with Some of the Imidazolium-Based Ionic Liquids. *J. Mol. Liq.* **2014**, *198,* 11–20.

(37) Zhang, M.; He, Z. Z.; Kang, R. X.; Ge, M. L. Thermodynamics and Activity Coefficients at Infinite Dilution for Organic Compounds in the Ionic Liquid 1-Hexyl-3-Methylimidazolium Chloride. *J. Chem. Thermodyn.* **2019**, *128,* 187–194.

(38) Yan, X. J.; Li, S. N.; Zhai, Q. G.; Jiang, Y. C.; Hu, M. C. Physicochemical Properties for the Binary Systems of Ionic Liquids [$C_n$mim]Cl + N,N-Dimethylformamide. *J. Chem. Eng. Data* **2014**, *59* (5), 1411–1422.

(39) Iguchi, M.; Hiraga, Y.; Sato, Y.; Aida, T. M.; Watanabe, M.; Smith, R. L. Measurement of High-Pressure Densities and Atmospheric Viscosities of Ionic Liquids: 1-Hexyl-3-Methylimidazolium Bis(Trifluoromethylsulfonyl)Imide and 1-Hexyl-3-Methylimidazolium Chloride. *J. Chem. Eng. Data* **2014**, *59* (3), 709–717.

(40) Yang, F.; Ma, Q.; Wang, X.; Liu, Z. Effect of Organic Solvents on Lowering the Viscosity of 1-Hexyl-3-Methylimidazolium Chloride. *J. Chem. Thermodyn.* **2017**, *113*, 358–368.

(41) Hazrati, N.; Abdouss, M.; Miran Beigi, A. A.; Pasban, A. A.; Rezaei, M. Physicochemical Properties of Long Chain Alkylated Imidazolium Based Chloride and Bis(Trifluoromethanesulfonyl)Imide Ionic Liquids. *J. Chem. Eng. Data* **2017**, *62* (10), 3084–3094.

(42) Vilas-Boas, S. M.; Teixeira, G.; Rosini, S.; Martins, M. A. R.; Gaschi, P. S.; Coutinho, J. A. P.; Ferreira, O.; Pinho, S. P. Ionic Liquids as Entrainers for Terpenes Fractionation and Other Relevant Separation Problems. *J. Mol. Liq.* **2021**, *323*, 114647.

(43) Yu, Y.-X.; Gong, Q.; Huang, L.-L. Measurement of Activity Coefficient at Infinite Dilution of Hydrocarbons in Sulfolane Using Gas−Liquid Chromatography. *J. Chem. Eng. Data* **2007**, *52* (4), 1459–1463.

(44) Mollmann, C.; Gmehling, J. Measurement of Activity Coefficients at Infinite Dilution Using Gas Liquid Chromatography. 5. Results for n-Me Thylacetamide, n,n-Dimethylacetamide, N-N-Dibutylformamide, and Sulfolane as Stationary Phases. *J. Chem. Eng. Data* **1997**, *42*, 35–40.

(45) Williams-Wynn, M. D.; Letcher, T. M.; Naidoo, P.; Ramjugernath, D. Activity Coefficients at Infinite Dilution of Organic Solutes in N-Formylmorpholine and N-Methylpyrrolidone from Gas-Liquid Chromatography. *J. Chem. Thermodyn.* **2013**, *61*, 154–160.

(46) Krummen, M.; Gmehling, J. Measurement of Activity Coefficients at Infinite Dilution in N-Methyl-2-Pyrrolidone and N-Formylmorpholine and Their Mixtures with Water Using the Dilutor Technique. *Fluid Phase Equilib.* **2004**, *215* (2), 283–294.

(47) Owe, S.; Yokoyama, K.; Nakamura, S. Vapor-Liquid Equilibrium Data for the System Acetone-Methanol Saturated with Salts. *J. Chem. Eng. Japan* **1969**, *2* (1), 1–4.

(48) Ghuge, P. D.; Mali, N. A.; Joshi, S. S. Effect of $CaCl_2$ and $ZnCl_2$ Salts on Isobaric Vapor-Liquid Equilibrium in Separation of the Azeotropic Mixture of Ethanol + Water. *Fluid Phase Equilib.* **2021**, *537*, 113000.

(49) Ge, Y.; Zhang, L.; Yuan, X.; Geng, W.; Ji, J. Selection of Ionic Liquids as Entrainers for Separation of (Water + Ethanol). *J. Chem. Thermodyn.* **2008**, *40* (8),

1248–1252.

(50) Zhao, J.; Dong, C. C.; Li, C. X.; Meng, H.; Wang, Z. H. Isobaric Vapor-Liquid Equilibria for Ethanol-Water System Containing Different Ionic Liquids at Atmospheric Pressure. *Fluid Phase Equilib.* **2006**, *242* (2), 147–153.

(51) Geng, W.; Zhang, L.; Deng, D.; Ge, Y.; Ji, J. Experimental Measurement and Modeling of Vapor-Liquid Equilibrium for the Ternary System Water + Ethanol + 1-Butyl-3-Methylimidazolium Chloride. *J. Chem. Eng. Data* **2010**, *55* (4), 1679–1683.

(52) Rajendran, M.; Renganarayanan, S.; Srinivasan, D. Salt Effect in Phase Equilibria: Effect of Dissolved Inorganic Salts on the Liquid-Liquid Equilibria of Benzene-2-Propanol-Water System and the Vapor-Liquid Equilibria of Its Constituent Binaries. *Fluid Phase Equilib.* **1989**, *50* (1–2), 133–164.

(53) Yin, L.; Li, Y.; Zhao, H.; Li, Q. L.; Wang, J.; Liu, F.; Xiao, L. N. Salts Effect on Isobaric Vapor-Liquid Equilibrium for the Azeotropic Mixture 2-Propanol + Water. *J. Chem. Eng. Data* **2019**, *64* (6), 2329–2340.

(54) Dongshun, D.; Yuzhen, Q.; Dengxiang, J. I.; Yun, G. E. Measurement and Modeling of Vapor-Liquid Equilibrium for Ternary System Water + 2-Propanol + 1-Butyl-3-Methylimidazolium Chloride. *Chinese J. Chem. Eng.* **2014**, *22* (2), 164–169.
